# Multimodal Raman and Fluorescence Lifetime Imaging: A New Analytical Approach for Resolving Timing-Dependent Metabolic Recovery in Endothelial Dysfunction

**DOI:** 10.1021/acs.analchem.6c01681

**Published:** 2026-07-27

**Authors:** Anna Pieczara, Julian Plitzko, Aleksandra Borek-Dorosz, Krzysztof Brzozowski, Marko Rodewald, Hyeonsoo Bae, Tobias Meyer-Zedler, Michael Schmitt, Jurgen Popp, Malgorzata Baranska

**Affiliations:** † Faculty of Chemistry, 37799 Jagiellonian University, Gronostajowa 2, 30-387 Krakow, Poland; ‡ Jagiellonian Centre for Experimental Therapeutics (JCET), 37799 Jagiellonian University, Bobrzynskiego 14, 30-348 Krakow, Poland; § Institute of Physical Chemistry (IPC) and Abbe Center of Photonics (ACP), 9378 Friedrich Schiller University Jena, Helmholtzweg 4, 07743 Jena, Germany; ∥ Leibniz Institute of Photonic Technology Jena, Albert-Einstein-Strasse 9, 07745 Jena, Germany; ⊥ Dipartimento di Fisica, Politecnico di Milano, Piazza Leonardo da Vinci 32, 20133 Milano, Italy

## Abstract

Endothelial dysfunction involves profound metabolic and biochemical disturbances that disrupt vascular homeostasis, yet temporal recovery patterns remain analytically underexplored. This study presents a high-content analytical platform integrating spontaneous Raman scattering, stimulated Raman scattering, coherent anti-Stokes Raman scattering, and fluorescence lifetime imaging microscopy to quantitatively map molecular alterations associated with endothelial dysfunction in vitro. The multimodal approach enables simultaneous assessment of lipid metabolism, biochemical composition, and cellular bioenergetics with subcellular spatial resolution. Distinct spectral biomarkers were identified, including diagnostic Raman bands at 701, 1748, 2884, and 3011 cm^–1^, complemented by nonspectral descriptors such as reduced cell volume and accelerated lipid droplet formation. Chemometric analysis utilizing principal component analysis, partial least-squares regression, and semiquantitative lipid profiling revealed a pronounced timing-dependent response to enalaprilat. Preventive treatment was found to preserve metabolic integrity, whereas post-treatment induced only partial restitution and generated a divergent metabolic trajectory. These findings demonstrate that early intervention leads to analytically distinguishable molecular states, underscoring the relevance of temporal profiling in endothelial diagnostics. By establishing a multimodal spectroscopic and chemometric framework, this work highlights the analytical power of integrative imaging for resolving subtle metabolic phenotypes and refining strategies for the objective evaluation of therapeutic efficacy.

## Graphical abstract

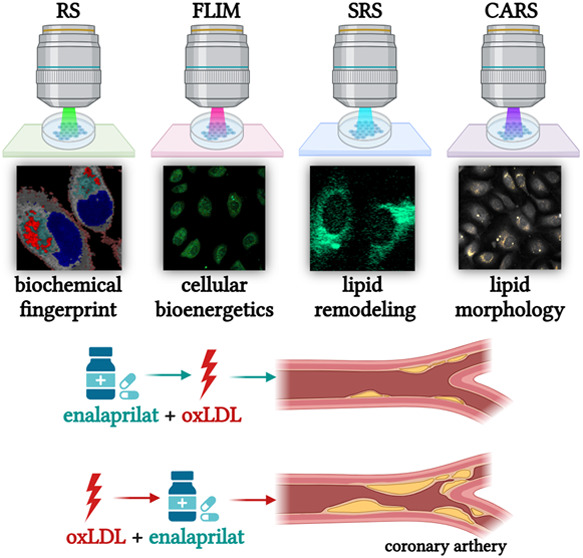

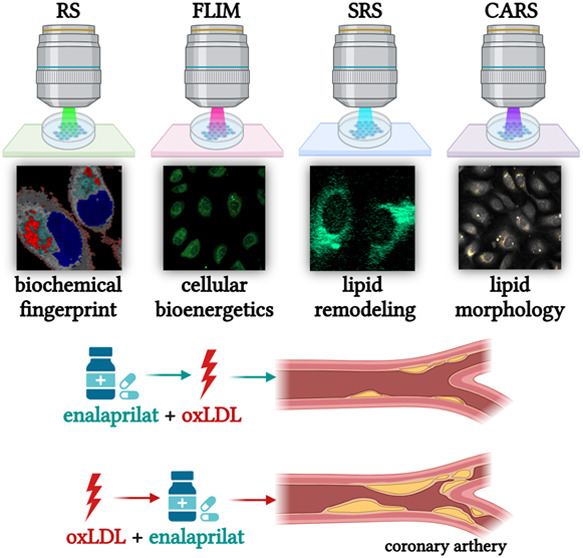

## Introduction

Cardiovascular diseases (CVD) remain the leading cause of mortality worldwide, emphasizing the urgent need for innovative diagnostic strategies and more effective therapeutic interventions.^1,2^ The vascular endothelium plays a central role in cardiovascular homeostasis by regulating vascular tone, permeability, inflammation, thrombosis, and redox balance.^3,4^ Endothelial cells (ECs) are highly metabolically active and respond dynamically to biochemical and mechanical stimuli. Despite their key role in vascular pathology, ECs remain underutilized as direct diagnostic and therapeutic targets.^5,6^

Endothelial dysfunction (ED) is a hallmark of early CVD and contributes to the progression of atherosclerosis, hypertension, diabetes, cancer, and neurodegenerative disorders.^7^ In atherosclerosis, ED is driven by chronic inflammation, oxidative stress, and lipid imbalance, leading to impaired nitric oxide bioavailability, increased vascular permeability, and enhanced leukocyte adhesion.^8–10^ The oxidized low-density lipoprotein (oxLDL) is a driver of these processes, accumulating within the arterial wall, and acts as a potent pro-inflammatory stimulus.^11,12^ OxLDL interacts with ECs primarily through the lectin-like oxLDL receptor (LOX-1), triggering intracellular signaling cascades that promote oxidative stress, mitochondrial dysfunction, and activation of the renin–angiotensin system.^13,14^ The binding of oxLDL to LOX-1 leads to the activation of angiotensin II type 1 (AT_1_R) receptors, the upregulation of adhesion molecules, and the recruitment of monocytes to the endothelium. These monocytes migrate into the subendothelial space, internalize oxLDL, and differentiate into foam cells, further amplifying inflammation and lipid accumulation.^12^ Angiotensin-converting enzyme inhibitors (ACEi) are widely used in the treatment of hypertension and heart failure and have demonstrated beneficial effects on endothelial function beyond blood pressure control.^15^ Enalapril, a prodrug converted to its active metabolite enalaprilat, belongs to the class of dicarboxylate-containing ACE inhibitors and has been shown to reduce oxidative stress, inflammation, and ED in clinical and experimental studies.^16,17^ However, it remains unclear whether ACE inhibition can reverse established ED or whether its primary benefit lies in preventing the onset of inflammatory damage.

Recent advances in imaging technologies provide unprecedented opportunities to investigate ED at the molecular and metabolic level. Spontaneous Raman spectroscopy (RS) enables label-free characterization of cellular composition, including lipids, proteins, and nucleic acids, with subcellular resolution.^18–20^ Nonlinear Raman techniques, such as stimulated Raman scattering (SRS) and coherent anti-Stokes Raman scattering (CARS), allow faster imaging with higher sensitivity and are particularly suited for visualizing lipid metabolism and lipid droplet (LD) dynamics.^21–24^ Fluorescence lifetime imaging microscopy (FLIM)^25,26^ complements Raman-based approaches by probing the metabolic state of cells through endogenous fluorophores such as FAD (flavin adenine dinucleotide) and NADH (nicotinamide adenine dinucleotide), which reflect the balance between glycolysis and oxidative phosphorylation (OXPHOS).^27,28^

While individual spectroscopic techniques have been applied to study endothelial biology, integrated multimodal approaches addressing both the biochemical composition and metabolic state remain insufficient. Moreover, the question of ED reversibility and the role of treatment timing have not been systematically addressed using molecularly resolved imaging. Therefore, the aim of this study was to identify molecular and metabolic markers of oxLDL-induced ED and to evaluate the extent to which these alterations can be prevented or reversed by enalaprilat by using a comprehensive spectroscopic approach.

## Materials and Methods

### Materials

Fetal bovine serum (FBS), phosphate-buffered saline (PBS), penicillin, streptomycin, fungizone, and oxLDL were purchased from Life Technologies (Thermo Fisher). Hydrocortisone, epidermal growth factor (EGF), and enalaprilat (EP) were obtained from Sigma–Aldrich.

### Cell Culture

Human coronary artery endothelial cells (HCECs) were cultured in EGM-2MV medium (Lonza) supplemented with 10% FBS, hydrocortisone (1 μg/mL), EGF (10 ng/mL), and antibiotics (streptomycin, penicillin, and fungizone, all at 1%). Cells were maintained at 37 °C in a humidified atmosphere of 5% CO_2_. For spectroscopic measurements, cells were seeded onto CaF_2_ slides at a density of 150 000 cells per slide and incubated for 24 h prior to treatment. Cells were treated with oxLDL (50 μg/mL, 6 h) to induce ED. Enalaprilat (5 μM, 1 h) was applied either before oxLDL incubation (pretreatment) or after oxLDL exposure (post-treatment). Untreated cells and cells treated with enalaprilat alone served as controls. Following treatment, cells were fixed with glutaraldehyde (2.5%) for 4 min, washed with PBS, and stored at 4 °C. All experiments were performed in three independent biological replicates.

### Spontaneous Raman Microscopy

Raman microspectroscopy was performed on a WITec confocal Raman microscope (WITec GmbH) operated via WITec Control software. Single-cell Raman imaging was performed with 532 nm laser excitation and a 60× water-immersion objective (Nikon Fluor Dic, NA = 1). For cell imaging, a pixel size of 0.6 μm and an integration time of 0.2 s were chosen. The mapped area was adjusted to the cell size. Up to 25 cells were imaged per sample in each biological replicate. The Raman spectrum of an oxLDL droplet on a CaF_2_ slide was collected with a 20× air objective (Zeiss EC Epiplan, NA = 0.45). The sample was excited at the maximum laser power (∼30 mW, at the sample), with 10 repetitions collected at an integration time of 0.5 s.

### Stimulated Raman Scattering

SRS measurements were performed on a Tandem Raman microscopy (TRAM) system with a picosecond *Lazurite* laser system (*Fluence* sp. z o.o., Poland), described in ref^29^. The Stokes pulses were centered at 1029 nm (maximum power of 450 mW). The pump beam was tuned in a range of 700–950 nm (average power of 100 mW). The Stokes beam underwent modulation at 4 MHz with an acousto-optic modulator (AOM). A Nikon Ti2 inverted microscope (Nikon Corp., Tokyo, Japan) was equipped with a 2-axis galvo scanner, a 40× air objective lens for focusing (Olympus UPLXAPO, NA = 0.95), a 50× water dipping objective lens for collecting (Nikon MRD07620, NA = 1.0), a SM1PD1 photodiode (Thorlabs) for signal detection, and an SR865A lock-in amplifier (Stanford Research Systems) for signal demodulation. This allows Stimulated Raman Loss (SRL) measurements. For the imaging of ECs, the power at the sample was 24 mW for the Stokes beam and 12 mW for the pump beam, utilized with a pixel dwell time of 300 μs and a pixel size of 500 nm × 500 nm.

### Coherent Anti-Stokes Raman Microscopy

CARS microspectroscopy was performed using a Leica confocal microscope (STELLARIS 8, Leica Microsystems GmbH) coupled with a laser system (picoEmerald) delivering 2 ps pulses, at an 80 MHz repetition rate (APE Angewandte Physik & Elektronik GmbH). The Stokes beam was centered at 1032 nm, and a pump was tuned in a range of 700–900 nm, (spectral bandwidth: 10 cm^–1^). The setup was equipped with a 63× oil-immersive objective (HC PL APO CS2, NA = 1.40). For cell imaging the pump pulse was set at 20 mW power (797.8 nm, at the sample) and the Stokes pulses at 30 mW (1032.6 nm, at the sample). The field of view was defined as 184.52 μm × 184.52 μm and was imaged with 10 frame averages. A three-dimensional dataset was measured with a *z*-resolution of 0.5 μm.

### Fluorescence Lifetime Imaging Microscopy

The FLIM experiments were performed with the experimental setup described in the previous section, “Coherent Anti-Stokes Raman Microscopy”. The two-photon excited autofluorescence (TPEF) was excited at 700 nm for NADH and at 850 nm for FAD. The laser power was set to 150 mW (20 mW at the sample) for both wavelengths. The field of view was defined as 184.52 μm × 184.52 μm. The pixel dwell time was 7.69 μs.

### Data Analysis and Preprocessing

The RS imaging data were analyzed with Project FIVE 5.1. The preprocessing included cosmic ray removal (CRR; filter size: 4; dynamic factor: 4) and background subtraction (BG; polynomial order: 3). A *k*-means cluster analysis (KMCA) was performed.

The integral intensities of selected Raman bands were obtained with the D method implemented in OPUS 7.0 software (Bruker) at 2930 (ν_str_(C–H) in CH_3_, lipids, and proteins), 2850 (ν_str_(C–H) in CH_2_, lipids), 1660 (ν_str_(C=C), amide I, lipids and proteins), 1440 (δ_def_(CH_3_, CH_2_) lipids and proteins), 1006 (phenylalanine ring breathing, proteins), and 720 cm^–1^ (ν­(N^+^(CH_3_)_3_) of choline, phospholipids) on averaged Raman spectra from the KMCA.^30–33^ The statistical significance of all identified changes was tested using an ANOVA model with post hoc Tukey’s test in OriginPro 9.1 (OriginLab Corporation, statistical significance: (***) *p* ≤ 0.001, (**) *p* ≤ 0.01, (*) *p* ≤ 0.05). The ANOVA analysis results are presented as a bar plot with the standard deviation (SD) indicated by boxes, the mean value is indicated by a line, the min and max values are indicated by whiskers, and the results of each spectrum are represented by dots. The data plots were created with Origin Pro 9.1.

The SRS image analysis was performed with Project FIVE 5.1. Ratiometric images were generated based on raw frames collected for lipids (2850 cm^–1^) and proteins (2930 cm^–1^). The protein frames were used to normalize the acquired frames, following the approach defined by Jamieson.^34^ The ratios of *I*_2850 cm^–1^_/*I*_2850 cm^–1^_ + *I*_2930 cm^–1^_ were calculated with the calculator option in Project FIVE 5.1. The results were plotted using OriginPro 9.1.

The three-dimensional CARS microspectroscopic data were analyzed using tailor-made analysis routines in FIJI (Figure S1).^35^ This approach enabled characterization of round structures with high signal intensity within cells. The resulting data was handled in OriginPro 2024.

1 $$I\left(t\right)=a_{1}\cdot e^{-t/\tau_{1}}+a_{2}\cdot e^{-t/\tau_{2}}$$

The FLIM data were analyzed within the internal software LAS X (Leica Microsystems GmbH). A square spatial binning of 3 and an automated peak thresholding were applied. For each dataset, a two-component exponential fit (eq 1) was performed. The resulting two-component data and the subsequent statistical analysis were processed using tailor-made routines in Python 3.8.10 and OriginPro 2024 (Figure S2).

### Chemometrics

Principal component analysis (PCA) was performed on the averaged Raman spectra obtained from the whole cell and/or on lipids utilizing Unscrambler X 10.3 software (CAMO Software AS.). The Raman spectra were preprocessed by smoothing (Savitzky–Golay, third-order polynomial, 17 or 21 pts), baseline correction, and unit vector normalization. The PCA was performed on the CH-stretching region (2830–3030 cm^–1^) and the fingerprint region (600–1800 cm^–1^, for cytoplasm). The variability in Raman spectra was presented in score plots, along with loadings. The data were presented using OriginPro 2021.

Partial least-squares discriminant analysis (PLS-DA) was performed using OPUS 7.0 software (Bruker Optik GmbH) to quantify differences between pre-treatment and post-treatment. The algorithm distinguished between the control and oxLDL Raman spectra in the CH stretching region (2830–3030 cm^–1^) using spectral pre-processing (constant offset elimination, multiplicative scattering correction, and internal standard correction). The models were presented in true versus predicted score plots along with the regression coefficients, reporting the Raman bands used for the differentiation process. The classification model was tested on unencountered Raman spectra from pre- and post-treatment samples. The classification result is presented as the sample number versus prediction with color-coded dots following the prediction.

## Results and Discussion

### Displaying the Path to Atherosclerosis: The Role of oxLDL in Endothelial Dysfunction

To establish reliable molecular markers of ED, HCECs were incubated with oxLDL (50 μg/mL, 6 h), a well-established in vitro model of inflammatory endothelial injury.^36,37^ OxLDL exposure is known to induce lipid uptake, oxidative stress, and metabolic disturbances characteristic of early atherogenesis, thereby providing a biologically relevant framework for spectroscopic analysis.

The Raman spectrum of oxLDL (Figure S3) was already discussed in detail in the literature and exhibits a characteristic lipid-dominated profile.^38^ A detailed band assignment of an oxLDL sample deposited on a CaF_2_ substrate is provided in Figure S3 and Table S1. Besides the typical stretching vibrations in the high-wavenumber region (ν_s_(−CH_3_), ν_as_(=CH_2_), and ν_s_(=CH_2_)) and higher fingerprint region (ν­(C=C), δ­(CH), α­(CH_2_)),^30,31^ five characteristic Raman bands at 607 (ρ­(CH_2_)), 702 (δ­(C=C) in cholesterol within the steroid ring), 719 (ν_s_(N^+^(CH_3_)_3_)), 881 (ν_as_(N^+^(CH_3_)_3_)), and 973 cm^–1^ (βCH) were observed.^33,39^ The presence of these bands provided a spectral benchmark for identifying oxLDL-derived signals within cells.

To objectively classify these biochemical features, KMCA was applied to hyperspectral RS datasets of oxLDL-treated HCECs. Pronounced biochemical heterogeneity was revealed at the single-cell level (Figure S4A). The resulting false-color maps revealed several spectrally distinct clusters corresponding to the cytoplasm, lipid-enriched cytoplasm, nucleus, membrane regions, and oxLDL-rich compartments. Importantly, oxLDL-associated clusters were predominantly localized to the cytoplasm rather than confined to the plasma membrane, indicating active internalization and intracellular processing of oxidized lipids. Representative mean spectra extracted from KMCA-defined clusters (Figure S4B) enabled detailed comparison of biochemical compositions. The spectra assigned to oxLDL-rich regions exhibited markedly enhanced intensity at 701 cm^–1^, confirming cholesterol enrichment, and 1748 cm^–1^ corresponding to C=O stretching associated with esterified lipids. Additionally, enhanced signals at 2884 and 3011 cm^–1^ were observed, corresponding to CH_2_ stretching and unsaturated lipid vibrations, respectively. In contrast, spectra from the control cytoplasm showed relatively lower lipid-associated intensities and a greater contribution of protein-related bands.

On the basis of these observations, four Raman bands (701, 1748, 2884, and 3011 cm^–1^) were selected as spectroscopic markers of oxLDL-induced ED. These bands collectively reflect cholesterol accumulation, esterified lipid species, and increased lipid unsaturation, all of which are hallmarks of inflammatory lipid remodeling in ECs.^30,31,33,39^

### Timing Matters: Raman-Based Dissection of Enalaprilat in Pre- and Post-Treatment Effects

After identifying the molecular signatures of oxLDL-induced ED, the impact of pharmacological intervention with enalaprilat was investigated by performing Raman microscopy on five experimental groups: control, enalaprilat alone, oxLDL, pretreatment (enalaprilat + oxLDL), and post-treatment (oxLDL + enalaprilat) (Figure 1A). Changes within the Raman spectrum of the lipid class extracted from the KMCA were investigated to assess the biochemical changes and the mechanism of action of oxLDL (Figure 1B).

**Figure 1:**
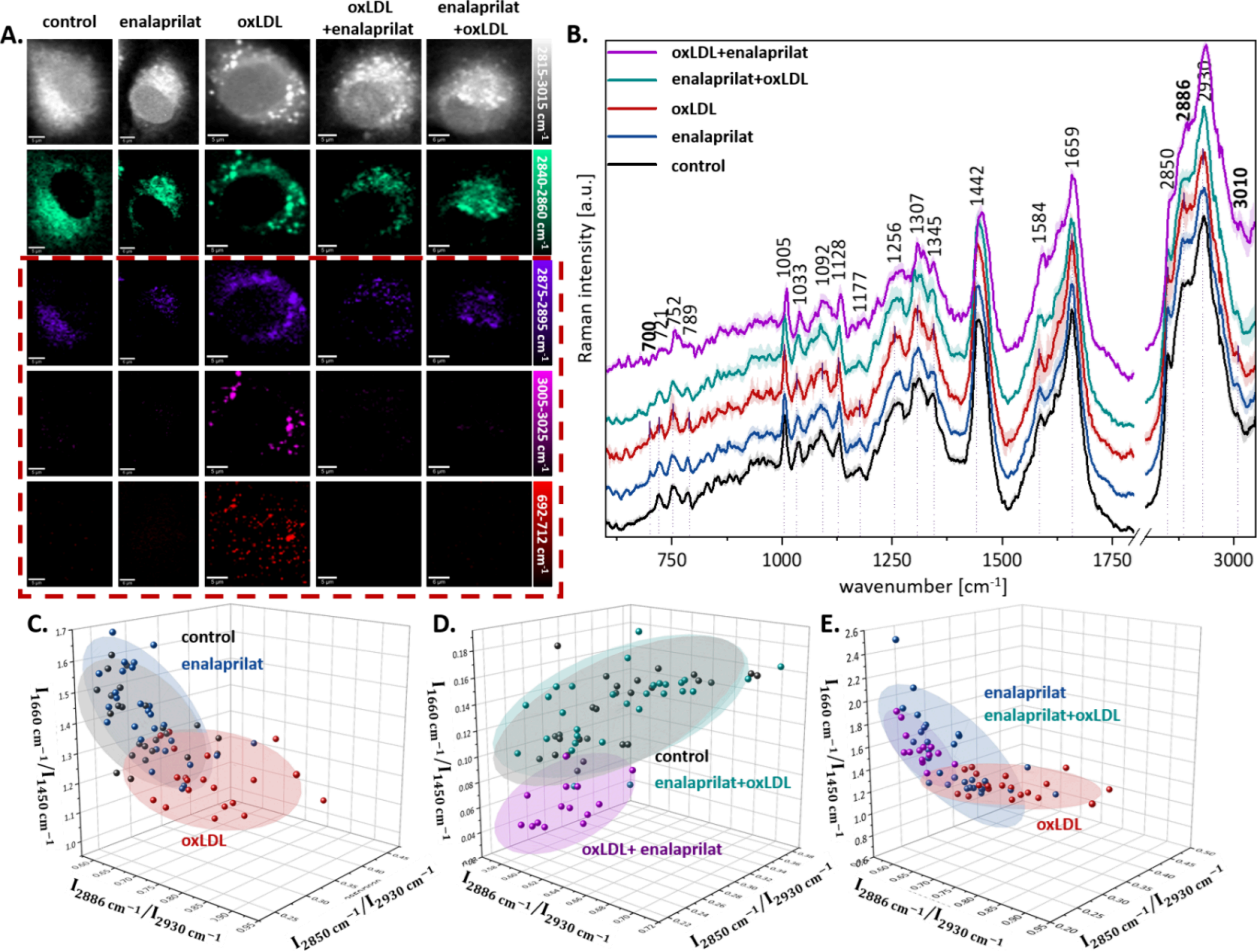
(A) Raman images of fixed HCEC. The Raman images were obtained by integrating the following spectral ranges: 2815–3015 (organic matter), 2840–2860 (unsaturated lipids), 2875–2895^1^ (saturated lipids, protein), 3005–3025 (saturated lipids), and 692–712 cm^–1^ (oxLDL). (B) The average Raman spectra of the lipid class from each sample. Raman spectra in the fingerprint region (500–1800 cm^–1^) were multiplied by 2.5 for better visualization. Scale bar = 5 or 6 μm. (C–E) 3D scatter plots illustrating lipidomic changes in HCECs. Each dot represents lipid-related changes in an individual cell. The *x*-axis shows oxLDL uptake by the cells (*I*_2886 cm^–1^_/*I*_2930 cm^–1^_), the *y*-axis represents cellular lipidity (*I*_2850 cm^–1^_/*I*_2930 cm^–1^_), and the *z*-axis indicates the degree of lipid unsaturation (*I*_1660 cm^–1^_/*I*_1450 cm^–1^_). Shaded areas are 70% confidence error ellipses.

For oxLDL-treated cells, the Raman signal intensity of the ED-associated marker bands increased, accompanied by notable morphological changes. Those morphological changes included reduced cell area, increased intracellular lipid content, and enhanced accumulation of lipid-rich spots, consistent with LDs. These changes are highlighted by increased Raman signal intensity in the spectral regions corresponding to saturated and unsaturated lipids as well as the emergence of strong oxLDL-specific signals at 701 cm^–1^. HCECs subjected to post-treatment with enalaprilat displayed Raman images that were largely indistinguishable from those of oxLDL-treated cells. Lipid-rich domains remained abundant, cell size remained reduced, and oxLDL-associated Raman bands persisted at high intensity (Figure 1B). The observation suggests that enalaprilat administration after oxLDL exposure does not reverse the established biochemical and structural alterations.

In contrast, pretreated cells exhibited a markedly different phenotype. Raman images of these cells closely resembled those of untreated controls, with reduced lipid signal intensity, absence of pronounced oxLDL-specific bands, and the preservation of cell morphology. These qualitative observations were supported by the quantitative analysis of Raman band ratios. A semiquantitative assessment of lipid-related Raman intensity ratios (Figure S4) revealed significant increases in the *I*_2886 cm^–1^_/*I*_2930 cm^–1^_ and *I*_2850 cm^–1^_/*I*_2930 cm^–1^_ ratios following oxLDL treatment, reflecting increased lipid content and LD accumulation. The *I*_1660 cm^–1^_/*I*_1450 cm^–1^_ ratio, indicative of lipid unsaturation, was also significantly elevated in oxLDL-treated cells.

Pre-treatment with enalaprilat effectively suppressed these changes, yielding ratios comparable to those of control cells. Post-treatment, however, resulted in only minor or statistically insignificant reductions. Collectively, these Raman-based analyses demonstrate that enalaprilat exerts a strong protective effect when administered prior to the oxLDL exposure but shows a limited capacity to reverse oxLDL-induced biochemical remodeling once established.

The results were further visualized using 3D scatter plots (Figure 1C–E) to highlight biochemical changes at the single-cell level. The axes represent cellular oxLDL uptake (*I*_2886 cm^–1^_/*I*_2930 cm^–1^_), lipid content (*I*_2850 cm^–1^_/*I*_2930 cm^–1^_), and lipid unsaturation (*I*_1660 cm^–1^_/*I*_1450 cm^–1^_). Control and enalaprilat-treated cells formed a closely overlapping cluster, indicating that enalaprilat did not induce lipid alterations or cellular damage. Pretreated cells clustered predominantly with control cells, whereas post-treated cells formed a separate group and exhibited substantial overlap with the oxLDL-treated cells.

Overall, the observed clustering patterns indicate that pretreatment is more effective than post-treatment in attenuating ED. However, neither strategy fully reversed the inflammatory phenotype in all of the cells.

### Metabolic Mosaic: Chemometric Tracking of Biochemical Changes Associated with oxLDL-Induced Endothelial Dysfunction

While univariate Raman band analysis provides valuable insights into specific biochemical alterations, the complexity of EDs necessitates multivariate approaches that capture global spectral patterns and subtle correlations among molecular features. To this end, PCA and PLS methods were applied to Raman spectra obtained from both the cytoplasmic and lipid-rich regions of HCECs to further elucidate the biochemical consequences of oxLDL exposure and pharmacological interventions.

PCA was performed on preprocessed Raman spectra in the C–H stretching region (2830–3030 cm^–1^), which is particularly sensitive to lipid and protein composition. To distinguish between global cellular changes and lipid-specific remodeling, separate PCA models were constructed from cytoplasmic data and from lipid-rich clusters identified by KMCA. The PCA score plot for cytoplasmic spectra (Figure 2A) shows separation between control and oxLDL-treated cells along a combination of PC1 and PC5 (loading presented on Figure 2B). Instead, lipid class discrimination (Figure 2C) appears more pronounced, while most cells with inflammation (oxLDL and pretreated cells) group on PC4+, whereas the others are more clustered around the negative site (PC4−), suggesting that the separation may occur along a component that explains less variance. Control cells formed a relatively compact cluster, reflecting biochemical homogeneity under physiological conditions. In contrast, oxLDL-treated cells displayed a broader distribution along PC1, consistent with increased biochemical heterogeneity arising from lipid uptake and oxidative stress. Pretreated cells clustered predominantly with control cells. However, the control group showed greater dispersion in the PCA score plot, indicating higher within-group variability than the more tightly clustered pretreated samples, which maintained a largely control-like biochemical profile. Post-treated cells were positioned closer to oxLDL-treated cells, indicating that oxLDL-induced biochemical alterations persisted despite pharmacological intervention. A similar clustering pattern was observed for the PCA of lipid-class spectra (Figure 2C). In this case, the separation between control and oxLDL-treated cells was even more pronounced, reflecting the strong impact of oxLDL on lipid composition. Pretreated cells again clustered near controls, whereas post-treated cells overlapped extensively with the oxLDL cluster. These PCA results indicate that oxLDL-induced biochemical remodeling affects both cytoplasmic and lipid-specific molecular signatures and that pretreatment with enalaprilat effectively suppresses these changes at a global biochemical level.

**Figure 2:**
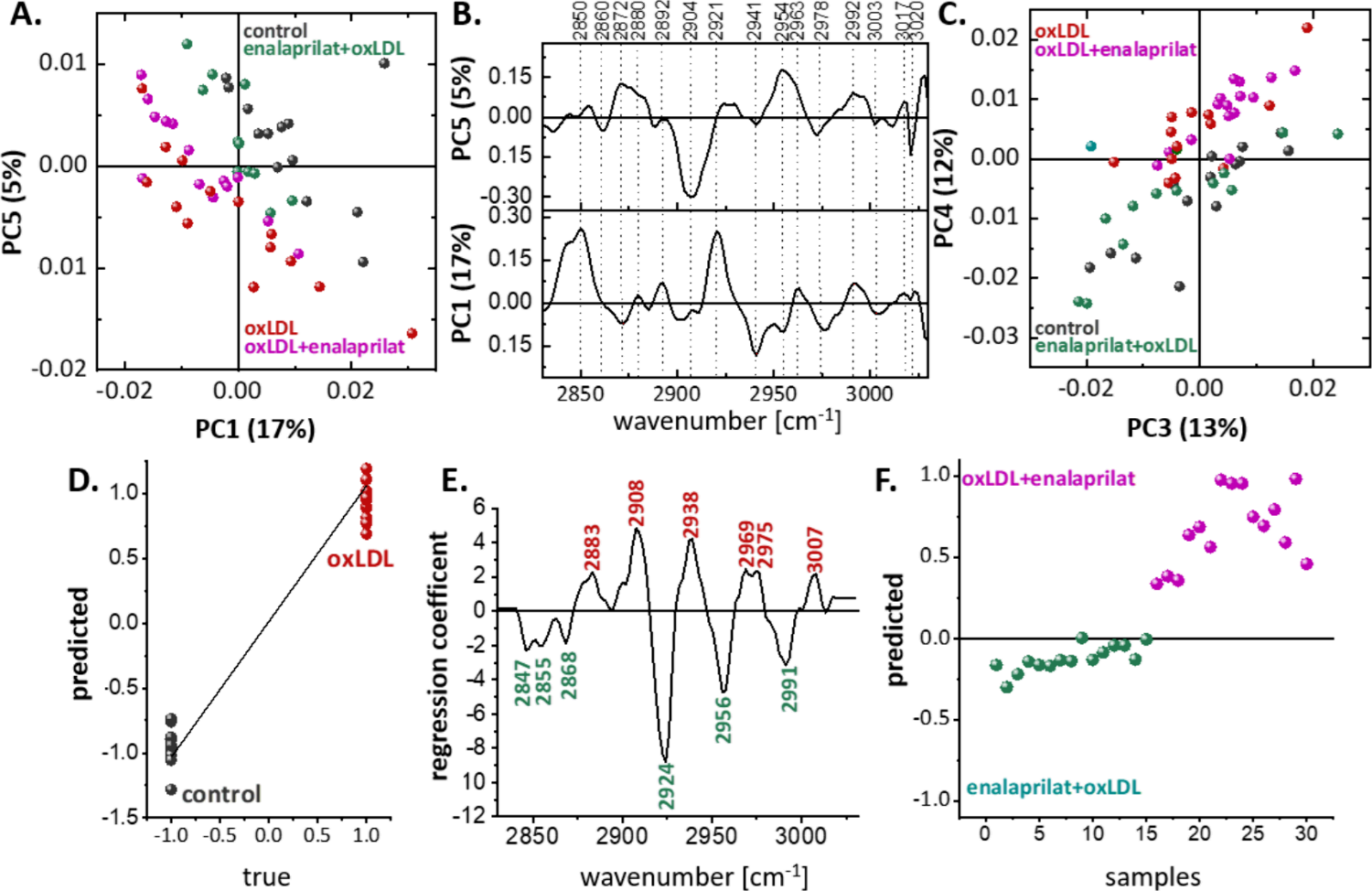
(A) PCA score plot and (B) loading plot performed in the C–H stretching region for the cytoplasm class. (C) PCA score plot for the lipid class. (D) PLS model (cytoplasm class) trained in the C–H stretching region (2830-3030 cm^-1^) on control and oxLDL samples. (E) The regression coefficient plot for the cytoplasm class. (F) Validation for the spectra from pre- and post-treated cells from the cytoplasm class.

To identify the molecular features underlying the observed clustering, PCA loading plots were examined (Figure 2B and Figure S6A). For cytoplasmic spectra, positive loadings on PC1 were dominated by Raman bands associated with unsaturated lipids and lipid esters, including bands at approximately 2883 and 3007 cm^–1^. These bands are indicative of increased lipid unsaturation and esterified lipid species, both of which are characteristic of the oxLDL-induced lipid remodeling. Negative loadings on PC1 were associated with bands related to saturated lipids and proteins, including features at 2924 and 2956 cm^–1^. These bands were more prominent in control cells, reflecting a balanced lipid-to-protein composition and lower levels of inflammatory lipid accumulation. In the lipid-class PCA, similar trends were observed, with unsaturated lipid-associated bands driving separation toward the oxLDL cluster and protein- and saturated lipid-associated bands contributing to the control cluster. These findings corroborate the univariate Raman band analyses and reinforce the interpretation that oxLDL exposure induces a shift toward unsaturated esterified lipid species within endothelial cells.

To achieve more robust discrimination between control and oxLDL-induced ED, partial least-squares regression (PLS-R) was applied to Raman spectra in the C–H stretching region (see Figure 2D and Figure S6B). In contrast to PCA, PLS-R is a supervised method that incorporates prior information about group membership, enabling identification of spectral features most strongly correlated with the inflammatory phenotype. A PLS-R model trained on control and oxLDL spectra achieved strong predictive performance, with a coefficient of determination (*R* ^2^) of approximately 0.97 and a low root-mean-square error of prediction (RMSEP). The high model performance indicates that the biochemical differences between control and inflamed cells are robust and reproducible. The regression coefficient plot derived from the PLS-R model (Figure 2E) provided further insight into the molecular basis of discrimination. Raman bands with positive coefficients, associated with oxLDL-treated cells, were dominated by features corresponding to unsaturated lipids (2883, 3007 cm^–1^), protein contributions (2908, 2938 cm^–1^), and esterified lipid species (2969, 2975 cm^–1^). In contrast, bands with negative coefficients, associated with control cells, were linked to vibrations of saturated lipids and proteins. These results highlight the central role of lipid unsaturation and esterification in defining the biochemical phenotype of oxLDL-induced ED.

To assess the extent to which enalaprilat pre- and post-treatment modulated the endothelial biochemical phenotype, a PLS-DA model was constructed. This model was trained exclusively on control and oxLDL spectra and then used to classify spectra from pretreated and post-treated cells that were not included in the training dataset. The PLS-DA classification results are presented in Figure 2F and Figure S6D. The majority of post-treated cells were classified as oxLDL-like, indicating that their biochemical profiles remained dominated by inflammatory lipid features. In contrast, pretreated cells were predominantly classified as control-like, although some dispersion was observed, reflecting biological variability.

Importantly, the classification confidence for pretreated cells was generally lower than that for control cells, suggesting that while pretreatment effectively suppresses major oxLDL-induced alterations, subtle biochemical differences persist. These findings are consistent with earlier RS, SRS, and CARS results and reinforce the conclusion that enalaprilat pretreatment preserves endothelial homeostasis, whereas post-treatment provides limited biochemical recovery.

### Multi-Method Lipidomics: CARS and SRS Decode Structural and Quantitative Lipid Remodeling in Endothelial Dysfunction

SRS and CARS microscopies were employed to further investigate lipid metabolism, LD formation, and morphological alterations associated with the oxLDL-induced ED. Nonlinear techniques based on the Raman effect offer a higher imaging speed and sensitivity than RS microscopy for the identification of ED markers. SRS microscopy was applied to visualize lipid and protein distributions in HCECs by selectively probing the CH_2_ stretching vibration at 2850 cm^–1^ (lipids) and the CH_3_ stretching vibration at 2930 cm^–1^ (proteins).

To minimize potential laser-induced effects, excitation powers were carefully optimized and kept as low as possible while preserving an adequate signal-to-noise ratio. The pump and Stokes powers at the sample were 20 mW and 30 mW for CARS imaging, and 12 mW and 24 mW for SRS imaging, respectively. Under these imaging conditions, no detectable signs of laser-induced photodamage were observed, including morphological alterations, local signal degradation, or signal instability during acquisition. As the samples were fixed, phototoxic effects associated with live-cell imaging were not applicable.^40–42^

Representative SRS images for control, enalaprilat-treated, oxLDL-treated, post-treated (oxLDL + enalaprilat), and pretreated (enalaprilat + oxLDL) cells are shown in Figure 3A. Qualitative inspection revealed pronounced differences in the lipid-associated signal intensity. Cells treated with oxLDL exhibited a markedly increased signal at 2850 cm^–1^, indicative of an elevated lipid content, frequently appearing as discrete, high-intensity spots consistent with LD accumulation. To account for variations in cell thickness, illumination conditions, and acquisition parameters, a ratiometric approach was employed.

**Figure 3:**
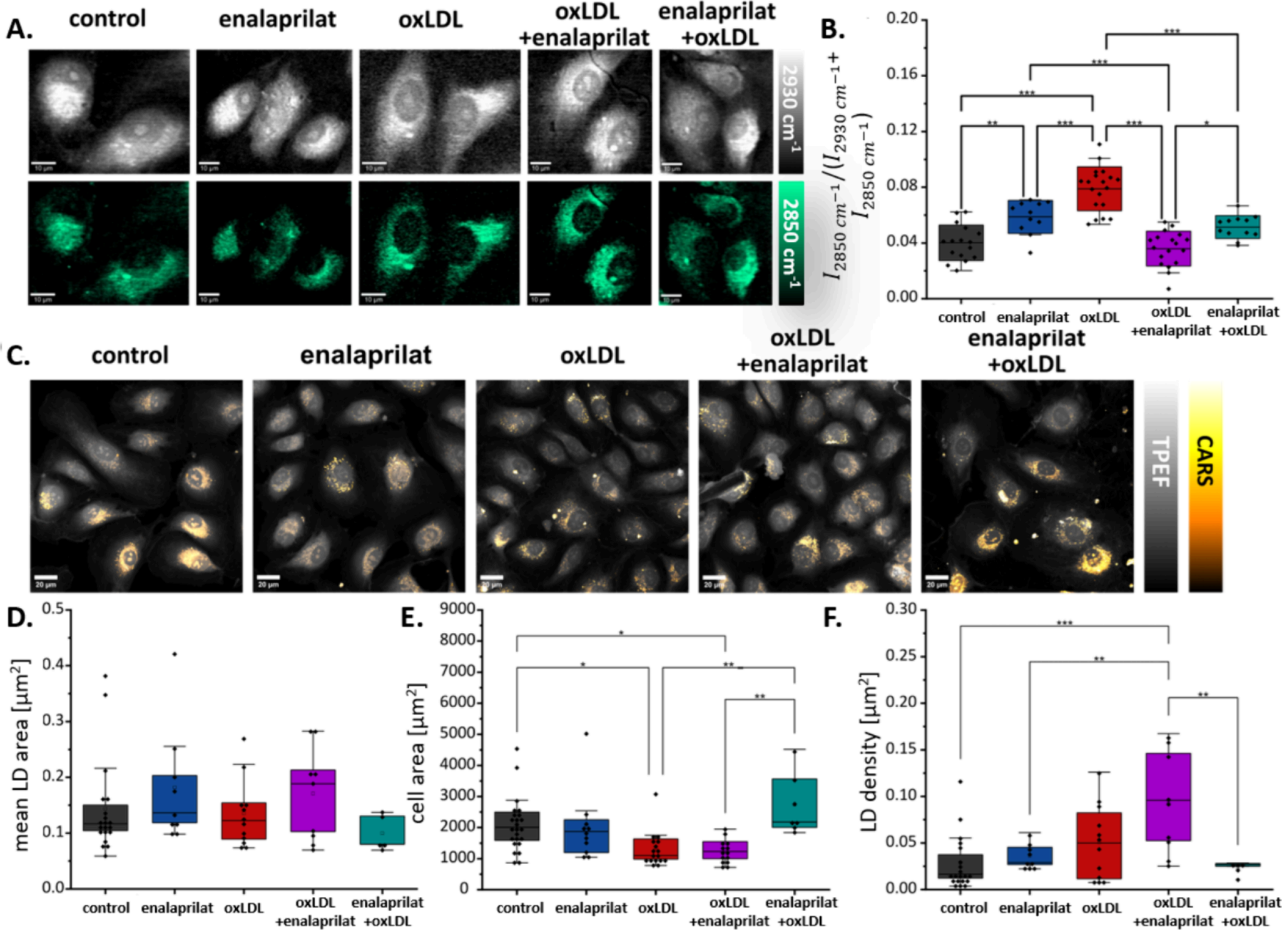
SRS and CARS markers of oxLDL-induced ED identified. (A) SRS images of fixed HCEC. (B) Quantitative ratiometric analysis of the SRS intensity ratio *I*_2850 cm^–1^_/(*I*_2850 cm^–1^_ + *I*_2930 cm^–1)^_. (C) CARS images of fixed HCEC. (D) The mean area of LDs within the cells, (E) the cell area of the imaged cells, and (F) the number of LDs normalized by the cell area are presented as box plots (mean ± SD, whiskers indicate minimum/maximum, line indicates the mean value). (***) *p* ≤ 0.001, (**) *p* ≤ 0.01, (*) *p* ≤ 0.05. Scale bar: 10 μm (SRS) and 20 μm (CARS).

The lipid signal at 2850 cm^–1^ was normalized to the combined lipid and protein signal using the ratio *I*_2850 cm^–1^_/(*I*_2850 cm^–1^_ + *I*_2930 cm^–1^_), following established methodologies for the semiquantitative assessment of cellular lipidicity.^34,43^ This normalization minimizes the influence of cell-to-cell variability and enables a robust comparison across samples. Quantitative analysis of the SRS ratiometric data (see Figure 3B, as well as Figure S7) demonstrated a statistically significant increase in lipidicity in oxLDL-treated cells compared with controls. Importantly, both pre- and post-treatment with enalaprilat yielded *I*_2850 cm^–1^_/(*I*_2850 cm^–1^_ + *I*_2930 cm^–1^_) ratios comparable to those of control cells. This indicates that at the level of the overall cellular lipid pool, enalaprilat attenuated oxLDL-induced lipid accumulation in both treatment settings. It should be emphasized, however, that this ratiometric approach reflects total cellular lipid content rather than lipids specifically derived from oxLDL, which may account for differences observed in analyses focused on oxLDL-associated lipid fractions. Importantly, despite their similarity to control levels, the pre- and post-treatment groups differed significantly, highlighting the impact of treatment timing on lipid remodeling dynamics.

In the utilized approach, SRS microscopy emphasizes quantitative information, while CARS microscopy focuses on three-dimensional investigations with an enhanced sensitivity to lipid-rich structures. The relationship among LD morphology, spatial distribution, and cellular morphology was thus investigated with CARS microscopy at approximately 2855 cm^–1^. Three-dimensional image stacks were collected for each experimental group, followed by maximum intensity projection to generate two-dimensional representations for quantitative analysis (Figure 3C**)**. Qualitative analysis of the segmented cell areas revealed a pronounced increase in the number of LDs in oxLDL-treated cells compared to controls. These LDs appeared as well-defined, spherical structures with high signal intensity, distributed throughout the cytoplasm.

Similar patterns were observed in post-treated cells, whereas pretreated cells exhibited LD distributions comparable to those of control cells, with fewer, more sparsely distributed droplets. For a quantitative analysis, several morphological parameters were extracted from the CARS datasets, including mean LD area, total cell area, and the number of LDs normalized to cell area (Figure 3D–F, Figure S8).

Analysis of the mean LD area (Figure 3D) revealed no statistically significant differences among experimental groups, indicating that oxLDL primarily affects the number of LDs rather than their size. In contrast, analysis of cell area revealed significant differences between groups (Figure 3E). Cells treated with oxLDL and post-treated cells exhibited a marked reduction in cell area compared to controls, consistent with endothelial contraction under inflammatory and oxidative stress conditions. Pretreated cells maintained cell areas comparable to those of control cells. Given the pronounced variability in cell area across experimental conditions, the LD number was normalized to cell area to obtain a more accurate measure of LD density (Figure 3F). Cells treated with oxLDL and post-treated cells exhibited a significant increase in LD density relative to controls, reflecting enhanced lipid storage within a reduced cellular volume. In contrast, pretreated cells did not show a significant increase in LD density compared to controls.

The combined SRS and CARS results provide complementary insights into lipid remodeling during the ED. SRS data demonstrate a global increase in cellular lipid content following oxLDL exposure, while CARS data revealed that this increase is primarily driven by an elevated number of LDs rather than changes in the individual droplet size. The observed reduction in the cell area under inflammatory conditions further amplifies lipid accumulation, leading to a higher LD density per unit cell area. This phenotype is characteristic of stressed ECs and is consistent with previous reports linking oxidative stress, cytoskeletal remodeling, and endothelial contraction to lipid-driven inflammation.

The chemometric analyses presented integrate seamlessly with univariate RS, SRS, and CARS findings. All approaches converge on the same biological interpretation: oxLDL induces profound lipid-driven biochemical remodeling in ECs and enalaprilat is most effective when administered prior to the inflammatory insult. Importantly, the SRS and CARS analyses converge that pretreatment with enalaprilat effectively prevents both lipid accumulation and cell contraction, preserving a control-like phenotype. These findings reinforce the concept that enalaprilat acts predominantly as a preventive agent, limiting the initiation of oxLDL-induced lipid uptake and remodeling rather than as a reparative agent capable of reversing established ED. The consistency across independent analytical methods underscores the robustness of the observed trends and emphasizes the critical importance of the timing of intervention in maintaining endothelial homeostasis.

### Metabolomics of Endothelial Dysfunction: FLIM Reveals Timing-Dependent Metabolic Reprogramming

While Raman-based techniques provide detailed insight into biochemical composition and lipid remodeling, they do not directly report on the dynamic metabolic state of cells. To complement the structural and compositional data, FLIM was employed to probe metabolic alterations associated with oxLDL-induced ED and to assess their modulation by enalaprilat. FLIM analysis focused on the endogenous fluorophores NADH and FAD, which serve as redox-active cofactors in cellular metabolism. Importantly, their fluorescence lifetimes depend on their protein-binding state, allowing discrimination between glycolytic and OXPHOS-dominated metabolic states.^44–46^ In cells, NADH exhibits a shorter lifetime in its free state (associated with glycolysis) and a longer lifetime when protein-bound (associated with OXPHOS), whereas FAD shows the opposite trend. By monitoring these changes, the cellular metabolic trajectory during ED and recovery can be precisely mapped.

An initial inspection of FLIM datasets was performed using mean photon arrival time images for NADH and FAD (Figure 4A). These images provided a qualitative overview of the lifetime distribution within the individual cells. Across all experimental groups, fluorescence lifetimes were largely homogeneous within single cells, indicating that metabolic alterations occurred globally rather than being confined to localized subcellular regions. Cells treated with oxLDL exhibited a visible shift toward shorter mean lifetimes for NADH, suggesting an increased proportion of free unbound NADH, typically associated with glycolytic metabolism. In contrast, control and pretreated cells displayed longer mean lifetimes, indicative of a higher fraction of protein-bound NADH and, consequently, greater mitochondrial involvement.

**Figure 4:**
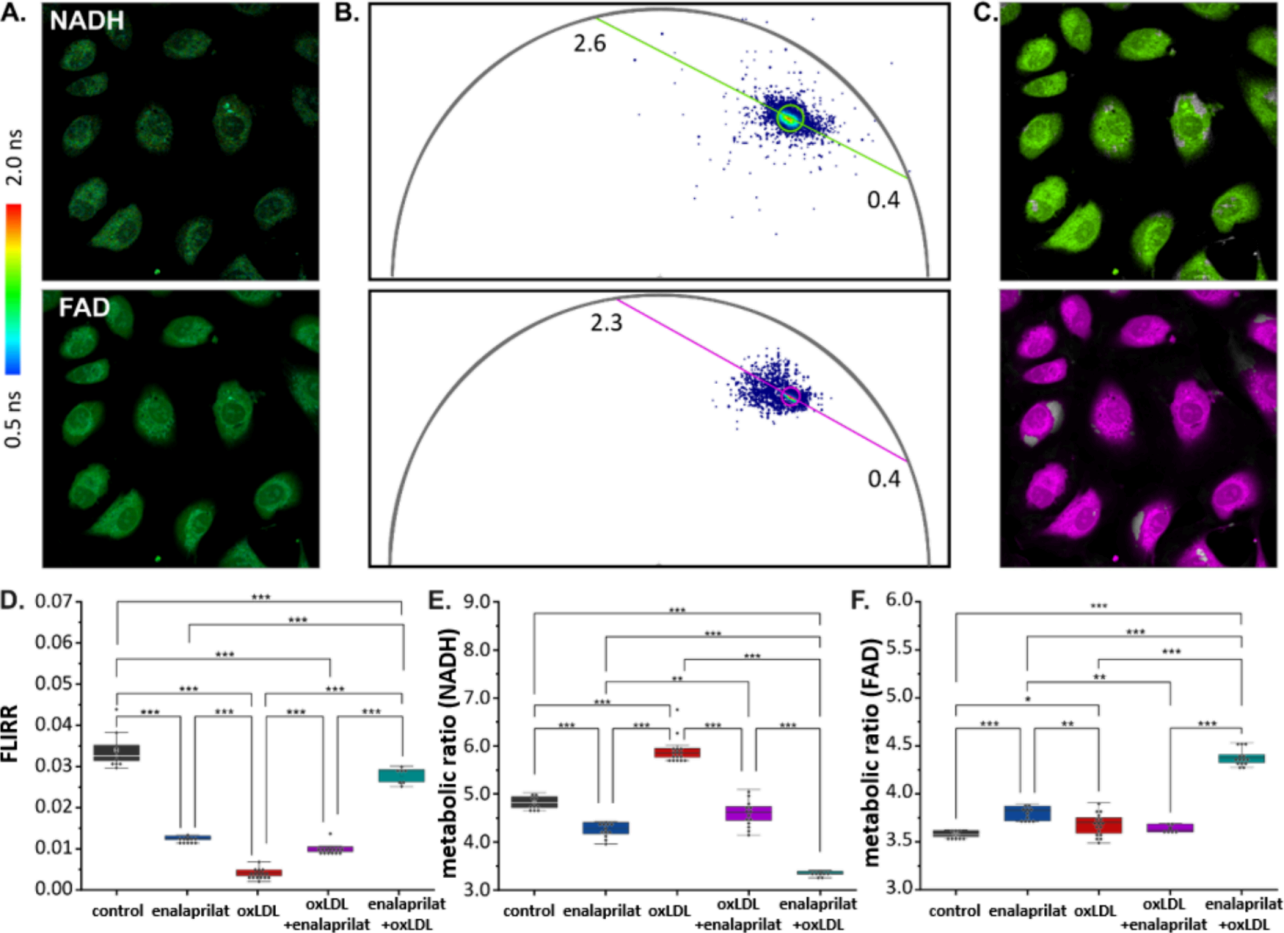
FLIM data analysis of biological triplicates of oxLDL-inflamed cells pre- and post-treated with enalaprilat. (A) Mean arrival time of the detected photons via TPE-FLIM of NADH and FAD within cells incubated with ED. (“Spectrum” LUT in the range of 0.5 to 2.0 ns.) (B) Phasor plots for NADH (green) and FAD (magenta) with a circular selection of the main distribution of data and the calculated two fluorescence lifetime components. (C) The projection of the circular selection within the phasor plot onto the greyscale intensity image. (D) FLIRR ratio for a variety of cell treatments. (E) The metabolic ratio for NADH. (F) The metabolic ratio for FAD. Results are presented as box plots (mean ± SD, whiskers indicate minimum/maximum, line indicates the mean value). (***) *p* ≤ 0.001, (**) *p* ≤ 0.01, (*) *p* ≤ 0.05.

To enable model-free visualization of fluorescence lifetime distributions, phasor plot analysis was applied to the FLIM datasets (Figure 4B). In the phasor representation, each pixel is projected into a two-dimensional space based on the Fourier transform of its decay curve, enabling intuitive identification of lifetime components. For both NADH and FAD, the phasor plots revealed distributions consistent with two dominant lifetime components: a short lifetime (∼0.4 ns) corresponding to the free, unbound state and a longer lifetime (∼2.3 ns for NADH and ∼2.6 ns for FAD) associated with protein-bound forms. Circular regions of interest were drawn around the main lifetime distributions, and the corresponding pixels were back-projected onto the fluorescence intensity images (Figure 4C). This back-projection confirmed that the analyzed lifetime signals originated from cellular regions, excluding contributions from background or extracellular fluorescence. For quantitative analysis, individual cells were segmented, and fluorescence decay curves were fitted after reasonable pixel binning utilizing a two-component exponential model. From the fitted amplitudes (*a*_1_ and *a*_2_), metabolic ratios were calculated as *a*_1_/*a*_2_ for NADH and FAD, along with the fluorescence lifetime imaging redox ratio (FLIRR), defined as *a*_2_(NADH)/*a*_1_(FAD)^44,45^ (see Figure S2 for details). Cells treated with oxLDL exhibited a significantly reduced FLIRR value compared to control cells (Figure 4D), indicating a metabolic shift toward glycolysis and reduced mitochondrial OXPHOS.

This shift is consistent with oxidative-stress-induced mitochondrial dysfunction and aligns with previous reports linking endothelial inflammation to impaired mitochondrial activity.^13,14^ The metabolic ratio for NADH (Figure 4E) was significantly increased in oxLDL-treated cells, while the metabolic ratio for FAD (Figure 4F) showed the opposite trend. A higher proportion of free NADH relative to protein-bound NADH supports the conclusion that glycolytic pathways are upregulated under inflammatory conditions, likely as a compensatory response to mitochondrial impairment. These results provide strong evidence of a glycolytic metabolic shift in oxLDL-induced ED.

Pretreatment with enalaprilat resulted in a pronounced preservation of metabolic parameters. FLIRR values in pretreated cells were significantly higher than those in oxLDL-treated cells and approached or exceeded control values. Similarly, NADH and FAD metabolic ratios indicated a shift toward an increased level of protein-bound NADH and enhanced OXPHOS. These findings suggest that enalaprilat pretreatment preserves mitochondrial function and prevents the metabolic reprogramming associated with ED. The observed metabolic profile is consistent with a reduced oxidative stress and improved redox balance.

Post-treated cells displayed a more complex metabolic response. FLIRR values and metabolic NADH/FAD ratios in this group were partially restored compared with oxLDL-treated cells, indicating some degree of metabolic normalization. However, these improvements were not accompanied by the reversal of lipid accumulation or structural alterations observed by Raman, SRS, and CARS microscopy. This discrepancy suggests that, while enalaprilat can mitigate certain metabolic shifts after the onset of ED, it is insufficient to decouple or reverse the established structural damage and lipid-rich phenotype once the inflammatory cascade is initiated. The FLIM results reveal a critical distinction between the metabolic and structural aspects of the ED. While post-treatment with enalaprilat appears to partially restore mitochondrial metabolic activity, it does not reverse lipid-driven structural remodeling or cell contraction. This dissociation suggests that metabolic compensation can occur independently of full biochemical or morphological recovery. In other words, ECs may regain aspects of metabolic flexibility without fully resolving the intracellular lipid accumulation and associated structural damage. In contrast, pretreatment with enalaprilat prevents both metabolic and structural alterations, reinforcing the concept that early pharmacological intervention is essential for preserving endothelial homeostasis. These integrated findings highlight that while metabolic pathways exhibit some plasticity, the structural signatures of endothelial injury, once established, are significantly more resistant to pharmacological reversal.

The multimodal imaging approach applied in this study was designed to provide complementary biochemical and metabolic information rather than to generate fully integrated datasets. FLIM and CARS measurements were performed on the same samples, enabling a comparative interpretation of the metabolic state and lipid distribution under consistent experimental conditions. In contrast, Raman and SRS imaging was conducted on separate, independently prepared samples following identical experimental protocols to ensure reproducibility and comparability across measurements. Due to differences in instrumentation and acquisition conditions, direct coregistration or pixel-wise correlation analysis was not performed. Instead, the results from individual modalities were interpreted in parallel, and their consistency across techniques was used to support the overall biological conclusions.

## Conclusions

This study provides an in-depth molecular and metabolic characterization of oxLDL-induced ED using a comprehensive, multimodal spectroscopic approach. By integrating RS, SRS, CARS, and FLIM, complementary yet convergent aspects of endothelial pathology were captured, spanning the biochemical composition, cellular structure, lipid remodeling, and metabolic state.

OxLDL exposure consistently induced a robust ED phenotype characterized by increased lipid accumulation, elevated lipid unsaturation, intracellular LD formation, and a reduced cell area. These structural and biochemical alterations were accompanied by pronounced metabolic reprogramming toward glycolysis, as evidenced by FLIM-derived changes in NADH and FAD fluorescence lifetimes and a reduction in the FLIRR ratio. Together, these features define a coherent molecular and metabolic signature of ED that aligns with known mechanisms of oxidative stress, mitochondrial impairment, and inflammatory activation in early atherogenesis.

A central outcome of this work is the demonstration that the biological reversibility of ED is strongly dependent on the timing of pharmacological intervention. Pretreatment with the ACE inhibitor enalaprilat effectively prevented oxLDL-induced biochemical, structural, and metabolic alterations. Cells exposed to enalaprilat prior to inflammatory stimulation retained a control-like phenotype across all investigated modalities, including Raman spectral fingerprints, LD abundance, cell morphology, and mitochondrial metabolic balance. These findings indicate that enalaprilat acts primarily as a protective agent, limiting oxLDL uptake and downstream lipid remodeling, while preserving endothelial metabolic homeostasis. In contrast, post-treatment with enalaprilat following oxLDL exposure resulted in only a partial recovery. While FLIM analysis revealed a degree of metabolic normalization, suggesting improved mitochondrial activity and redox balance, microspectroscopic analyses demonstrated persistent lipid accumulation, an elevated LD density, and a reduced cell area. This dissociation between metabolic and structural recovery highlights a critical aspect of ED: restoration of metabolic parameters does not necessarily equate to the reversal of lipid-driven structural remodeling. Once intracellular lipid reorganization and cell contraction are established, these alterations appear to be largely resistant to pharmacological reversal under the conditions investigated.

Importantly, this apparent discrepancy between metabolic and structural readouts is not contradictory but rather underscores the multifaceted nature of the ED. The data suggest that metabolic flexibility can be partially regained even in the presence of persistent lipid pathology, reflecting cellular compensation rather than a true recovery. This finding underscores the need for integrated analytical strategies when evaluating therapeutic efficacy as reliance on a single biomarker class may overestimate functional restoration. Beyond the specific biological insights into ED and ACE inhibition, this study demonstrates the value of advanced spectroscopic techniques as powerful tools for mechanistic investigation rather than as mere imaging modalities. Raman-based methods provide sensitive, label-free detection of lipid remodeling and biochemical composition, while FLIM adds a functional dimension by directly probing the cellular metabolism. The convergence of these independent techniques strengthens the robustness of the conclusions and enables a more nuanced interpretation of the complex cellular phenotypes.

In summary, the results presented here indicate that oxLDL-induced ED is biologically preventable but only partially reversible once lipid remodeling and structural changes have occurred. Early pharmacological intervention is therefore critical for preserving the endothelial integrity and metabolic balance. These findings have direct implications for the timing of therapeutic strategies targeting vascular inflammation and highlight the importance of early detection and intervention in cardiovascular diseases. The integrated spectroscopic framework established in this work provides a solid foundation for future studies aimed at refining diagnostic markers, elucidating drug mechanisms of action, and ultimately improving strategies for the prevention and management of ED.

## Supplementary Material



## Data Availability

Raw measurement data from the conducted research are available at 10.57903/UJ/1BX7TX. Based on these data, the figures presented in this publication were created.

## Notes

Detailed workflows for CARS and FLIM measurements, oxLDL standard spectrum, and additional supporting analyses (PDF)

## ABBREVIATIONS

ACEi: angiotensin-converting enzyme inhibitors
AT1: angiotensin II type 1 receptor
CARS: coherent anti-Stokes Raman scattering
CVD: cardiovascular diseases
EC: endothelial cell
ED: endothelial dysfunction
ETC: electron transfer chain
FAD: flavin adenine dinucleotide
FAs: fatty acids
FLIM: fluorescence lifetime imaging microscopy
FLIRR: fluorescence lifetime imaging redox ratio
HCA: hierarchical cluster analysis
HCEC: human coronary artery endothelial cells
KMCA: k-means cluster analysis
LD: lipid droplet
LOX- 1: lectin-like oxLDL receptor
NADH: nicotinamide adenine dinucleotide
oxLDL: oxidized low-density lipoprotein
OXPHOS: oxidative phosphorylation
PCA: principal component analysis
PLS-DA: partial least-squares discriminant analysis
RS: spontaneous Raman scattering
SRS: stimulated Raman scattering
TPEF: two-photon excited fluorescence
α: scissoring
β: bending
δ: deformation
ν: stretching (ν_s_, symmetric; ν_as_, asymmetric)
ρ: rocking
τ: twisting

## References

[1] Chapman N., Thomas E. E., Tan J. T. M., Inglis S. C., Wu J. H. Y., Climie R. E., Picone D. S., Blekkenhorst L. C., Wise S. G., Mirabito Colafella K.
M., Calkin A. C., Marques F. Z. (2022). A Roadmap of Strategies to Support Cardiovascular Researchers:
From Policy to Practice. Nat. Rev. Cardiol..

[2] Roth G. A., Mensah G. A., Johnson C. O., Addolorato G., Ammirati E., Baddour L. M., Barengo N. C., Beaton A., Benjamin E. J., Benziger C. P. (2020). Global Burden of Cardiovascular
Diseases and Risk Factors, 1990–2019: Update From the GBD 2019
Study. J. Am. College Cardiol..

[3] Galley H. F., Webster N. R. (2004). Physiology of the Endothelium. Br. J. Anaesth..

[4] Aird W. C. (2007). Phenotypic
Heterogeneity of the Endothelium: I. Structure, Function, and Mechanisms. Circ. Res..

[5] Jansen, F. ; Nickenig, G. ; Werner, N. Extracellular Vesicles in Cardiovascular Disease. Circ. Res. 2017, 120 1649–1657, 10.1161/CIRCRESAHA.117.310752. 28495995

[6] Martin-Ventura, J. L. ; Roncal, C. ; Orbe, J. ; Blanco-Colio, L. M. Role of Extracellular Vesicles as Potential Diagnostic and/or Therapeutic Biomarkers in Chronic Cardiovascular Diseases. Front. Cell Dev. Biol. 2022, 10.3389/fcell.2022.813885. PMC8827403 35155428

[7] Rajendran P., Rengarajan T., Thangavel J., Nishigaki Y., Sakthisekaran D., Sethi G., Nishigaki I. (2013). The Vascular
Endothelium and Human Diseases. Int. J. Biol.
Sci..

[8] Ross R. (1999). Atherosclerosis
— An Inflammatory Disease. New England
Journal of Medicine.

[9] Matsuura E., Hughes G. R. V., Khamashta M. A. (2008). Oxidation
of LDL and Its Clinical
Implication. Autoimmun. Rev..

[10] Stocker R., Keaney J. F. (2004). Role of Oxidative
Modifications in Atherosclerosis. Physiol. Rev..

[11] Galle J., Hansen-Hagge T., Wanner C., Seibold S. (2006). Impact of Oxidized
Low Density Lipoprotein on Vascular Cells. Atherosclerosis.

[12] Jiang H., Zhou Y., Nabavi S. M., Sahebkar A., Little P. J., Xu S., Weng J., Ge J. (2022). Mechanisms of Oxidized LDL-Mediated
Endothelial Dysfunction and Its Consequences for the Development of
Atherosclerosis. Front. Cardiovasc. Med..

[13] Yamamoto K., Kakino A., Takeshita H., Hayashi N., Li L., Nakano A., Hanasaki-Yamamoto H., Fujita Y., Imaizumi Y., Toyama-Yokoyama S., Nakama C., Kawai T., Takeda M., Hongyo K., Oguro R., Maekawa Y., Itoh N., Takami Y., Onishi M., Takeya Y., Sugimoto K., Kamide K., Nakagami H., Ohishi M., Kurtz T. W., Sawamura T., Rakugi H. (2015). Oxidized LDL (OxLDL) Activates the
Angiotensin II Type 1 Receptor by Binding to the Lectin-like OxLDL
Receptor. FASEB J..

[14] Takahashi T., Huang Y., Yamamoto K., Hamano G., Kakino A., Kang F., Imaizumi Y., Takeshita H., Nozato Y., Nozato S., Yokoyama S., Nagasawa M., Kawai T., Takeda M., Fujimoto T., Hongyo K., Nakagami F., Akasaka H., Takami Y., Takeya Y., Sugimoto K., Gaisano H. Y., Sawamura T., Rakugi H. (2021). The Endocytosis
of Oxidized LDL via the Activation of the Angiotensin II Type 1 Receptor. iScience.

[15] Tiefenbacher C. P., Friedrich S., Bleeke T., Vahl C., Chen X., Niroomand F. (2004). ACE Inhibitors and Statins Acutely
Improve Endothelial
Dysfunction of Human Coronary Arterioles. Am.
J. Physiol. Heart Circ. Physiol..

[16] Cleary J. D., Taylor J. W. (1986). Enalaprilat: A New
Angiotensin Converting Enzyme Inhibitor. Drug
Intell. Clin. Pharm..

[17] Faisal M., Cawello W., Laeer S. (2021). Clinical Pharmacokinetics
of Enalapril
and Enalaprilat in Pediatric Patients—A Systematic Review. Front. Pediatr..

[18] Mariani, M. M. ; Deckert, V. Raman Spectroscopy: Principles, Bene.Ts, and Applications. In Methods in Physical Chemistry; Wiley-VCH Verlag GmbH & Co. KGaA: Weinheim, Germany, 2012; Vol. 1, pp 419–444, 10.1002/9783527636839.ch13.

[19] Nowakowska A. M., Pieczara A., Korona W., Borek-Dorosz A., Adamczyk A., Dawiec P., Leszczenko P., Orleanska J., Machalska E., Orzechowska B., Orzechowska S., Brzozowski K., Krynicka W., Majzner K., Malek K., Baranska M. (2025). The Raman Map of the Human Cell. Anal. Chem..

[20] Krafft C., Popp J. (2015). The Many Facets of
Raman Spectroscopy for Biomedical Analysis. Anal. Bioanal. Chem..

[21] Evans C. L., Xie X. S. (2008). Coherent Anti-Stokes Raman Scattering
Microscopy: Chemical
Imaging for Biology and Medicine. Annual Review
of Analytical Chemistry.

[22] Camp C. H., Cicerone M. T. (2015). Chemically Sensitive
Bioimaging with Coherent Raman
Scattering. Nat. Photon..

[23] Wang H. W., Fu Y., Huff T. B., Le T. T., Wang H., Cheng J. X. (2009). Chasing
Lipids in Health and Diseases by Coherent Anti-Stokes Raman Scattering
Microscopy. Vib. Spectrosc..

[24] Brzozowski K., Matuszyk E., Pieczara A., Firlej J., Nowakowska A. M., Baranska M. (2022). Stimulated Raman Scattering
Microscopy in Chemistry
and Life Science - Development, Innovation, Perspectives. Biotechnol. Adv..

[25] Datta R., Heaster T. M., Sharick J. T., Gillette A. A., Skala M. C. (2020). Fluorescence
Lifetime Imaging Microscopy: Fundamentals and Advances in Instrumentation,
Analysis, and Applications. J. Biomed. Opt..

[26] Suhling K., Hirvonen L. M., Levitt J. A., Chung P. H., Tregidgo C., Le Marois A., Rusakov D. A., Zheng K., Ameer-Beg S., Poland S., Coelho S., Henderson R., Krstajic N. (2015). Fluorescence Lifetime Imaging (FLIM): Basic Concepts
and Some Recent Developments. Medical Photonics.

[27] Yaseen M. A., Sutin J., Wu W., Fu B., Uhlirova H., Devor A., Boas D. A., Sakadžić S. (2017). Fluorescence
Lifetime Microscopy of NADH Distinguishes Alterations in Cerebral
Metabolism in Vivo. Biomed. Opt. Express.

[28] Cao R., Wallrabe H. K., Periasamy A. (2020). Multiphoton
FLIM Imaging of NAD­(P)­H
and FAD with One Excitation Wavelength. J. Biomed.
Opt..

[29] Brzozowski K., Pieczara A., Nowakowska A. M., Korona W., Orzechowska B., Firlej J., Wislocka-Orlowska A., Baranska M. (2025). Tandem Raman Microscopy :
Integration of Spontaneous and Coherent Raman Scattering Offering
Data Fusion Analysis to Improve Optical Biosensing. Optica.

[30] Czamara K., Majzner K., Pacia M. Z., Kochan K., Kaczor A., Baranska M. (2015). Raman Spectroscopy of Lipids: A Review. J. Raman Spectrosc..

[31] Rygula A., Majzner K., Marzec K. M., Kaczor A., Pilarczyk M., Baranska M. (2013). Raman Spectroscopy of Proteins: A Review. J. Raman Spectrosc..

[32] Kuhar N., Sil S., Verma T., Umapathy S. (2018). Challenges in Application of Raman
Spectroscopy to Biology and Materials. RSC Adv..

[33] Krafft C., Neudert L., Simat T., Salzer R. (2005). Near Infrared Raman
Spectra of Human Brain Lipids. Spectrochim.
Acta A Mol. Biomol. Spectrosc..

[34] Jamieson L. E., Wetherill C., Faulds K., Graham D. (2018). Ratiometric Raman Imaging
Reveals the New Anti-Cancer Potential of Lipid Targeting Drugs. Chem. Sci..

[35] Schindelin J., Arganda-Carreras I., Frise E., Kaynig V., Longair M., Pietzsch T., Preibisch S., Rueden C., Saalfeld S., Schmid B., Tinevez J. Y., White D. J., Hartenstein V., Eliceiri K., Tomancak P., Cardona A. (2012). Fiji: An Open-Source
Platform for Biological-Image Analysis. Nat.
Methods.

[36] Chiu T. H., Ku C. W., Ho T. J., Tsai K. L., Yang Y. D., Ou H. C., Chen H. I. (2023). Schisanhenol Ameliorates OxLDL-Caused
Endothelial Dysfunction by Inhibiting LOX-1 Signaling. Environ. Toxicol..

[37] Su, Z. ; Wang, J. ; Xiao, C. ; Zhong, W. ; Liu, J. ; Liu, X. ; Zhu, Y. Z. Functional Role of Ash2l in OxLDL Induced Endothelial Dysfunction and Atherosclerosis. Cell. Mol. Life Sci. 2024, 81 (1), 10.1007/s00018-024-05130-5. PMC10821849 38280036

[38] Stiebing C., Schmölz L., Wallert M., Matthäus C., Lorkowski S., Popp J. (2017). Raman Imaging of Macrophages Incubated
with Triglyceride-Enriched OxLDL Visualizes Translocation of Lipids
between Endocytic Vesicles and Lipid Droplets. J. Lipid Res..

[39] Hanlon E. B., Manoharan R., Koo T. W., Shafer K. E., Motz J. T., Fitzmaurice M., Kramer J. R., Itzkan I., Dasari R. R., Feld M. S. (2000). Prospects for in Vivo Raman Spectroscopy. Phys. Med. Biol..

[40] Fu Y., Wang H., Shi R., Cheng J.-X. (2006). Characterization
of Photodamage in Coherent Anti-Stokes Raman Scattering Microscopy. Opt. Express.

[41] Meyer T., Bergner N., Krafft C., Akimov D., Dietzek B., Popp J., Bielecki C., Romeike B. F. M., Reichart R., Kalff R. (2011). Nonlinear Microscopy,
Infrared, and Raman Microspectroscopy for Brain
Tumor Analysis. J. Biomed. Opt..

[42] Zhang, X. ; Dorlhiac, G. ; Landry, M. P. ; Streets, A. Phototoxic Effects of Nonlinear Optical Microscopy on Cell Cycle, Oxidative States, and Gene Expression. Sci. Rep. 2022, 12 (1), 10.1038/s41598-022-23054-7. PMC9637160 36335145

[43] Pieczara A., Borek-Dorosz A., Buda S., Tipping W., Graham D., Pawlowski R., Mlynarski J., Baranska M. (2023). Modified Glucose as
a Sensor to Track the Metabolism of Individual Living Endothelial
Cells - Observation of the 1602 Cm–1 Band Called “Raman
Spectroscopic Signature of Life”. Biosens.
Bioelectron..

[44] Schaefer P. M., Kalinina S., Rueck A., von Arnim C. A. F., von Einem B. (2019). NADH Autofluorescence—A Marker on Its Way to
Boost Bioenergetic Research. Cytometry, Part
A.

[45] Kalinina S., Freymueller C., Naskar N., von Einem B., Reess K., Sroka R., Rueck A. (2021). Bioenergetic Alterations
of Metabolic Redox Coenzymes as Nadh, Fad and Fmn by Means of Fluorescence
Lifetime Imaging Techniques. Int. J. Mol. Sci..

[46] Barroso M., Monaghan M. G., Niesner R., Dmitriev R. I. (2023). Probing Organoid
Metabolism Using Fluorescence Lifetime Imaging Microscopy (FLIM):
The next Frontier of Drug Discovery and Disease Understanding. Adv. Drug Delivery Rev..

