# Supplementary material for: Multimodal Raman and Fluorescence Lifetime Imaging: A New Analytical Approach for Resolving Timing-Dependent Metabolic Recovery in Endothelial Dysfunction
Source: Anal Chem. 2026 Jul 27;98(31):22800–11. doi: 10.1021/acs.analchem.6c01681 (PMC13470981; doi:10.1021/acs.analchem.6c01681)
Supplement: Supplementary file 1 [file ac6c01681_si_001.pdf]

Supplementary Information

Multimodal Raman and Fluorescence Lifetime Imaging: A New Analytical Approach for Resolving Timing-Dependent Metabolic Recovery in Endothelial Dysfunction

Anna Pieczara[1,2,*], Julian Plitzko[3,4], Aleksandra Borek-Dorosz [1,4], Krzysztof Brzozowski[2], Marko Rodewald[3,4,5], Hyeonsoo Bae[3,4], Tobias Meyer-Zedler[3,4], Michael Schmitt[3,4], Jurgen Popp[3,4*], Malgorzata Baranska[1,2*]

[1] *Faculty of Chemistry, Jagiellonian University, Gronostajowa 2, 30-387 Krakow, Poland*
[2] *Jagiellonian Centre for Experimental Therapeutics (JCET), Jagiellonian University, Bobrzynskiego 14, 30-348 Krakow, Poland*
[3] *Institute of Physical Chemistry (IPC) and Abbe Center of Photonics (ACP), Friedrich Schiller University Jena, Helmholtzweg 4, 07743 Jena, Germany*
[4] *Leibniz Institute of Photonic Technology Jena, Albert-Einstein-Strasse 9, 07745 Jena, Germany*
[5] *Dipartimento di Fisica, Politecnico di Milano, Piazza Leonardo da Vinci 32, 20133 Milano, Italy*

Correspondence: anna.pieczara@uj.edu.pl, m.baranska@uj.edu.pl, juergen.popp@uni-jena.de

Table of contents

Fig. S1. ImageJ-based workflow of coherent anti-Stokes Raman scattering (CARS) microspectroscopy data treatment and analysis.
Fig. S2. Leica LAS X and Python workflow of two-photon excited fluorescence lifetime imaging microscopy (TPEF-FLIM) data treatment and analysis.
Fig. S3. Raman spectrum of oxLDL.
Table S1. Raman bands assignments for the Raman spectrum of oxLDL.
Fig. S4. KMCA of representative HCAEC incubated with oxLDL.
Fig. S5. Raman markers of oxLDL-induced ED.
Fig. S6. Chemometric analysis of spectra from the lipid class of cells treated with enalaprilat and oxLDL**.**
Fig. S7. Ratiometric Raman imaging of intracellular lipid distribution.
Fig. S8. Results of LD characterization with CARS microscopy.

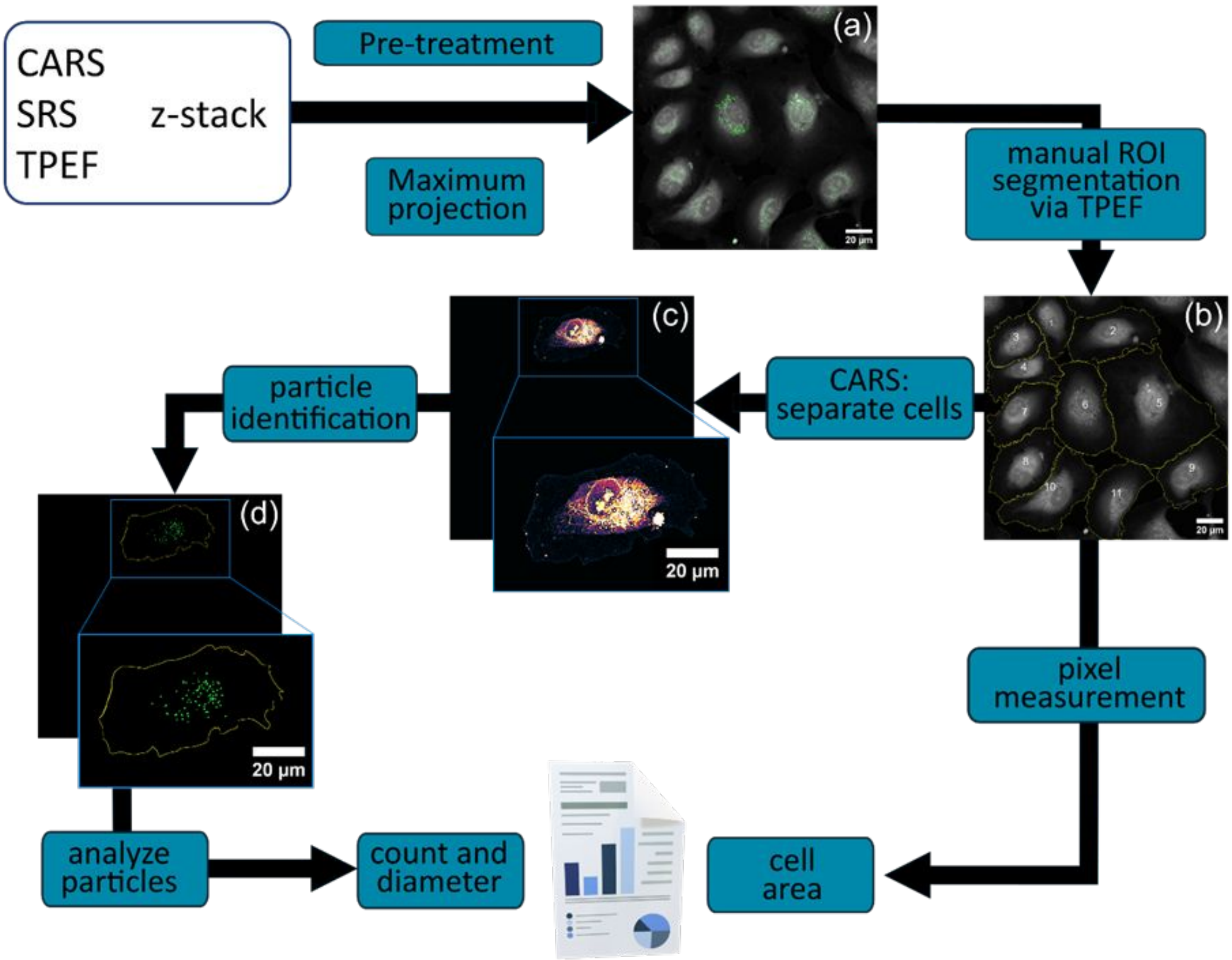

Fig. S1. ImageJ-based workflow of coherent anti-Stokes Raman scattering (CARS) microspectroscopy data treatment and analysis. *The three-dimensional data sets were pretreated layer-wise (Rigid correction and Gaussian filter) and projected into a* ***(a)*** *two-dimensional image by a maximum projection. Algorithm-based separation was not applicable therefore manual rois were created* ***(b)*** *as mask of the cell area. The cell area was measured. After separating the individual cells* ***(c)*** *a tailor-made routine of typical operations was utilized to identify and analyze the dot-like structure within each cell. The maximum positions are marked in green (d).*

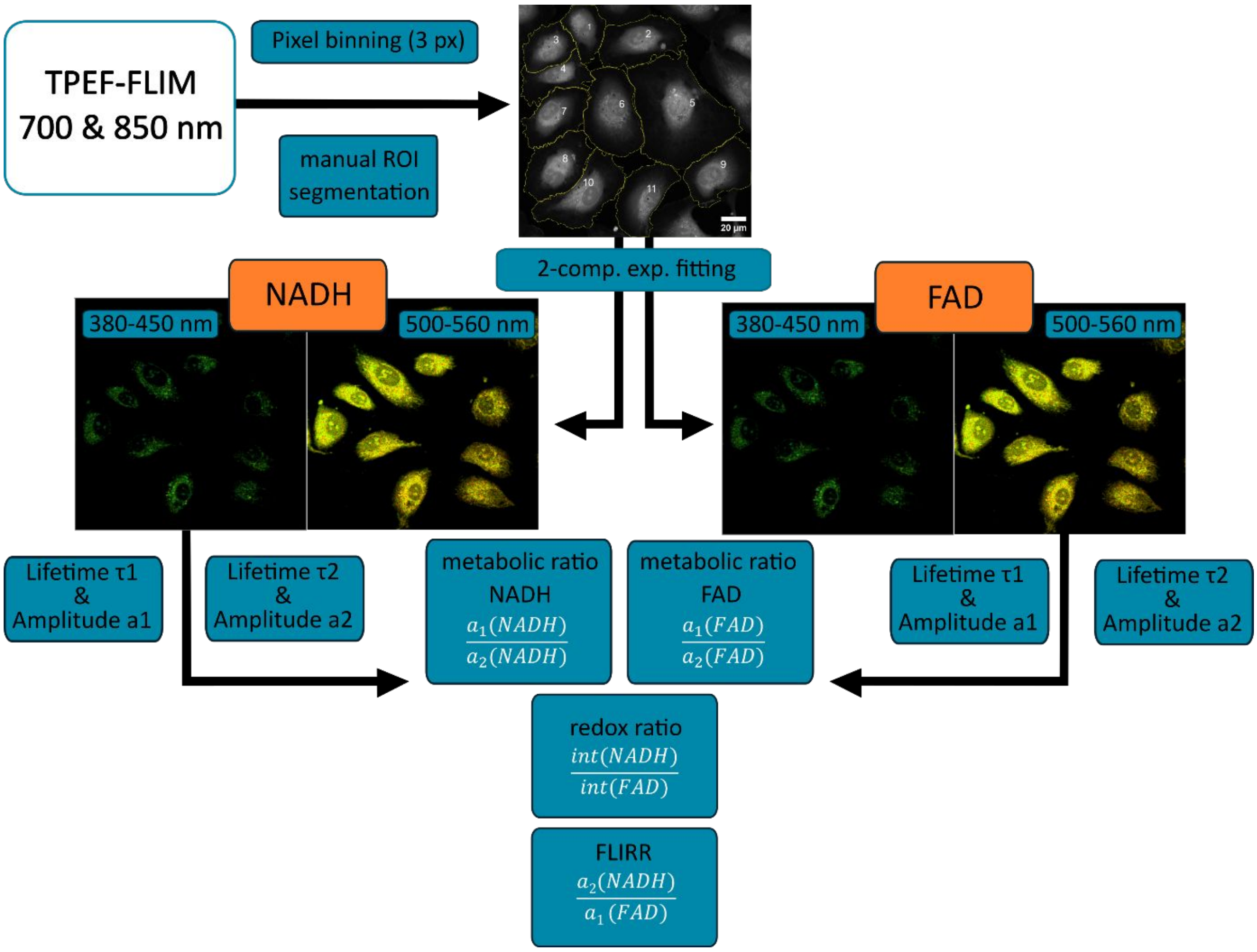

Fig. S2. Leica LAS X and Python workflow of two-photon excited fluorescence lifetime imaging microscopy (TPEF-FLIM) data treatment and analysis. *The fluorescence lifetime data is binned for 3 px within LAS X. Manual ROIs are created for every cell. Two-component exponential fitting is performed for all data sets. For the 700 nm excitation for NADH the amplitudes and lifetimes of the 380-450 nm detection channel were read out. For the 850 nm excitation for FAD the amplitudes and lifetimes of the 500-560 nm detection channel were read out. The metabolic ratio is calculated for every excitation wavelength by dividing the two amplitudes. The redox ratio is calculated by dividing the channel intensities for the chosen detection channels. FLIRR is calculated by dividing the amplitudes connected to the protein-bound form.*

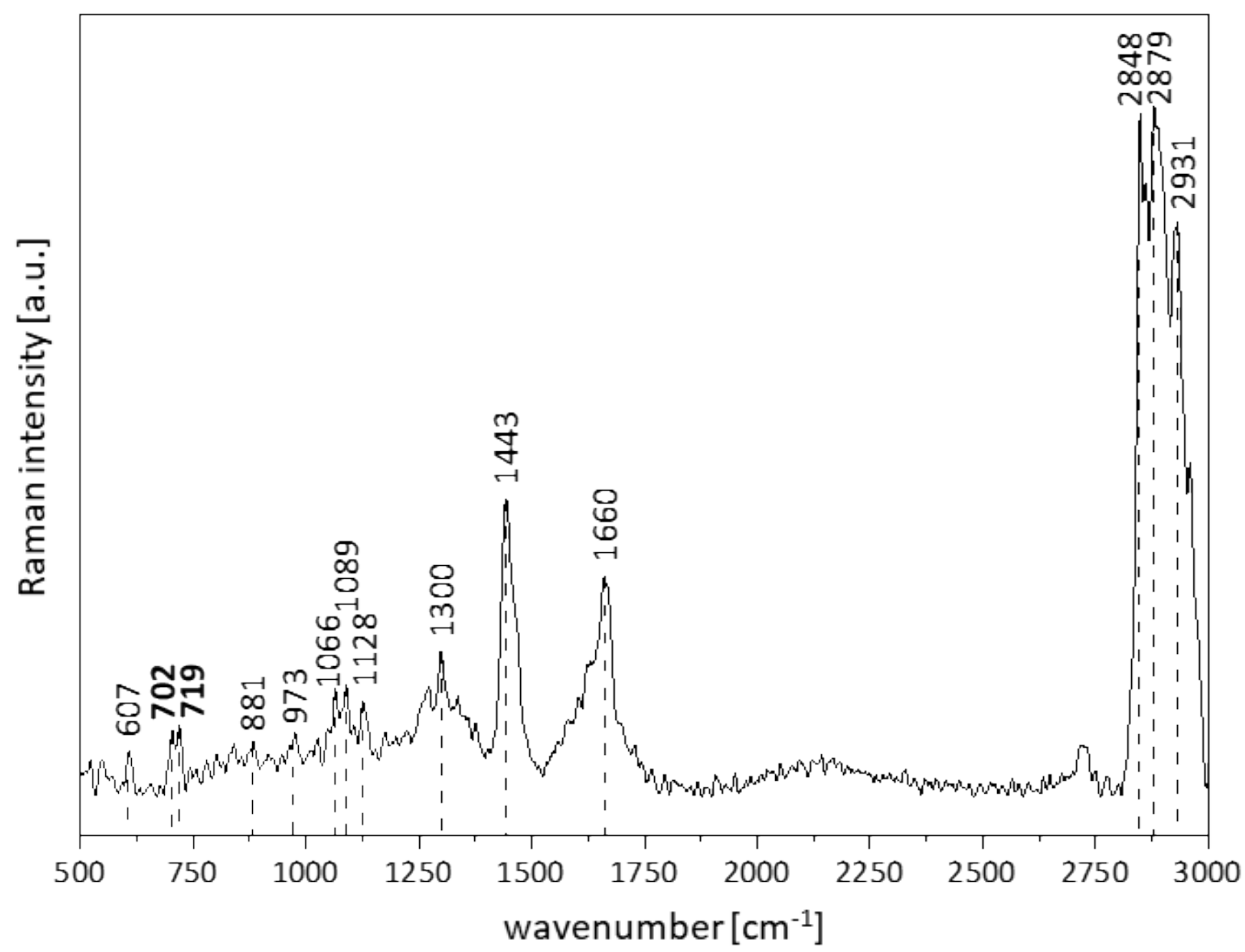

Fig. S3. *Raman spectrum of oxidized Low-density Lipoprotein (oxLDL).*

Table S1*: Raman band assignments for the Raman spectrum of oxLDL.*

Wavenumber [cm^{-1}]	Band assignment	Reference
607	$\rho(CH_2)$	
702	δ(C=C) in cholesterol	
719	$\nu_s(N^+(CH_3)_3)$	
881	$\nu_{as}(N^+(CH_3)_3)$	
973	β(CH)	
1066	ν(C-C)	
1089	$\nu_s(PO^{2-})$, ν(C-C)	
1128	$\nu_{as}(PO^{2-})$, ν(C-C)	[30, 31, 33, 39]
1272	δ(=CH)	
1300	$\tau(CH_2)$	
1443	$\alpha(CH_2/CH_3)$	
1660	ν(C=C)	
2848	$\nu_{as}(=CH_2)$	
2879	$\nu_{as}(=CH_2)$	
2931	$\nu_s(=CH_3)$	

Abbreviations: α – scissoring; τ – twisting; ν – stretching (s – symmetric; as – asymmetric), β – bending, δ – deformation; ρ – rocking;

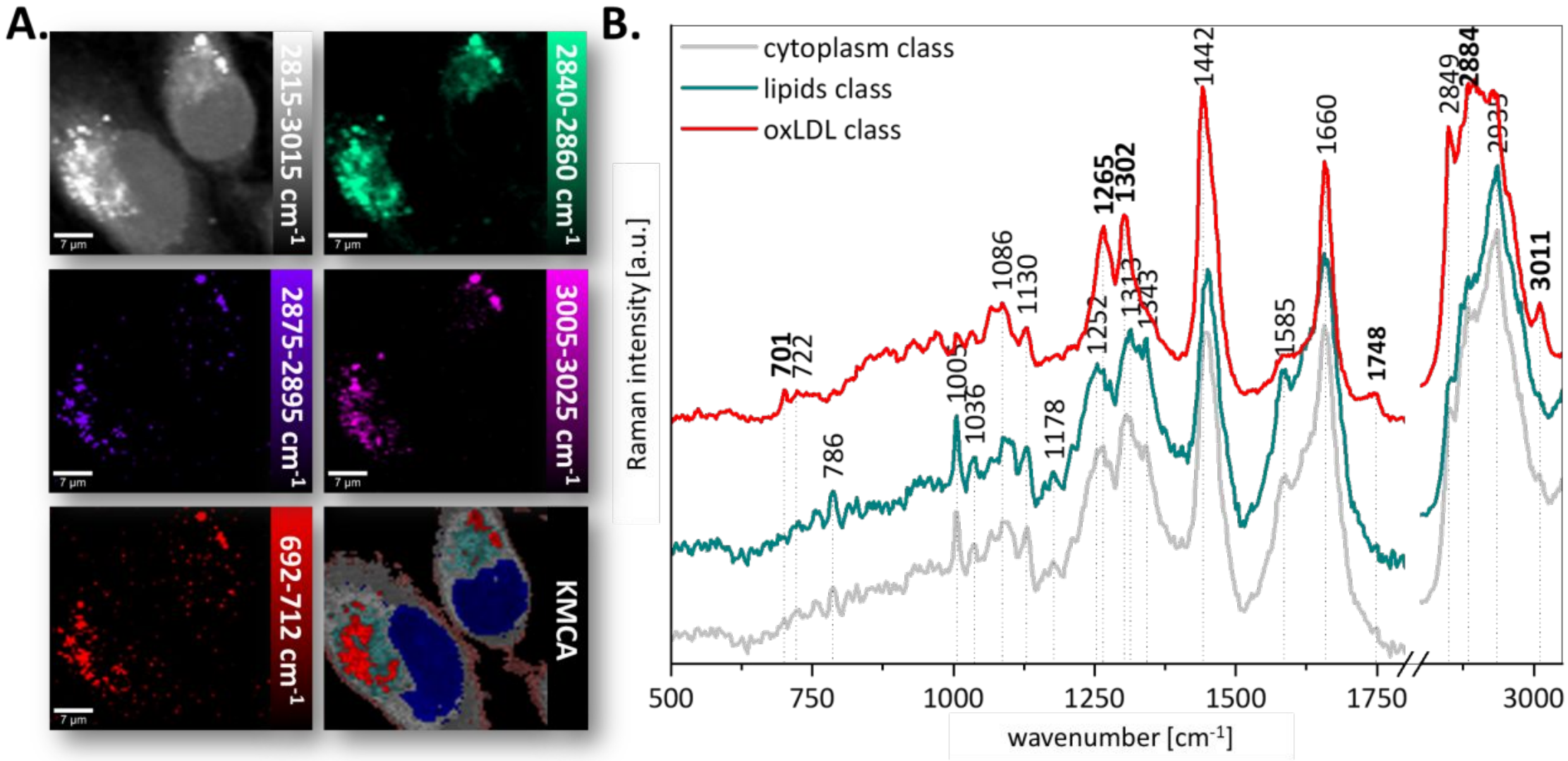

Fig. S4. KMCA of representative HCAEC incubated with oxLDL. A. *Raman images of fixed HCAEC incubated with oxLDL (50 µg/mL, 6 h) obtained by the integration of Raman bands in the regions: 2815 – 3015 cm^{-1} (organic matter), 2840 – 2860 cm^{-1} (unsaturated lipids), 2875 – 2895 cm^{-1} (saturated lipids, protein), 3005 – 3025cm^{-1} (saturated lipids), and 692 – 712 cm^{-1} (oxLDL), with corresponding false-color KMCA maps [unsaturated lipids (green), saturated lipids (red), nucleus (blue), cytoplasm (grey), membrane (brown), background (black)].* ***B.*** *The Raman spectra represent the classes of cytoplasm (grey), cytoplasm with a high lipid content (green), and cytoplasm with a high oxLDL content (red). Raman spectra in the fingerprint region (500 – 1800 cm^{-1}) were multiplied by 2.5 for better visualization. Bands characteristic of oxLDL-induced inflammation have been bolded. Scale bar: 7 µm.*

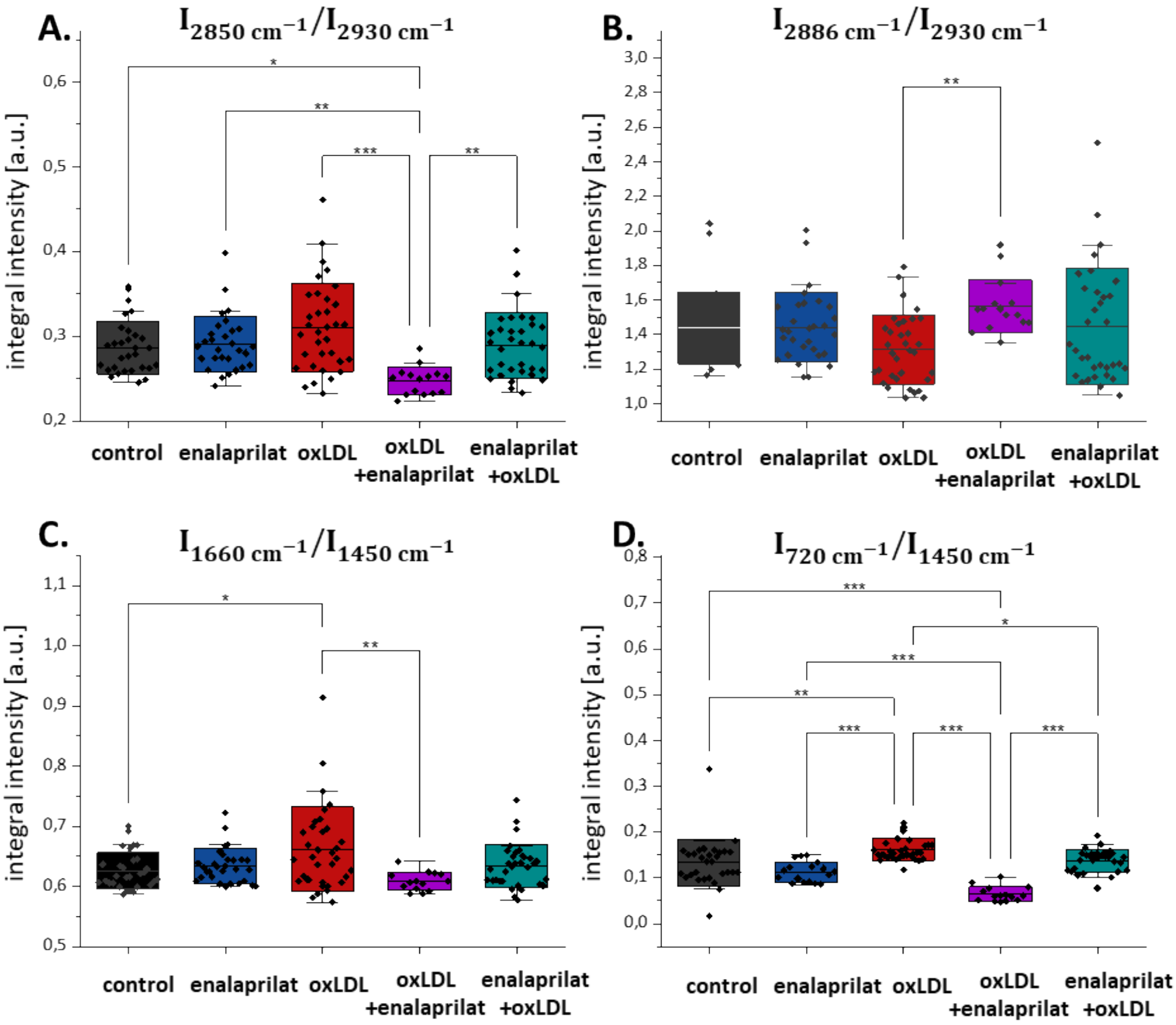

Fig. S5. Raman markers of oxLDL-induced ED. A. *Quantitative analysis of the level of unsaturation calculated based on the integral intensity* ratio $I_{2850\,cm^{-1}}/I_{2930\,cm^{-1}}$ *for average single-cell spectra of whole cytoplasm class,* ***B.*** *The integral intensity ratio* $I_{2886\,cm^{-1}}/I_{2930\,cm^{-1}}$. ***C.*** *The integral intensity ratio* $I_{1660\,cm^{-1}}/I_{1450\,cm^{-1}}$. ***D.*** *The integral intensity ratio* $I_{720\,cm^{-1}}/I_{1450\,cm^{-1}}$. *Results are presented as box plots (mean ± SD, whiskers indicate minimum/maximum, line indicates the mean value), ***p≤ 0.001, **p≤ 0.01, *p≤ 0.05.*

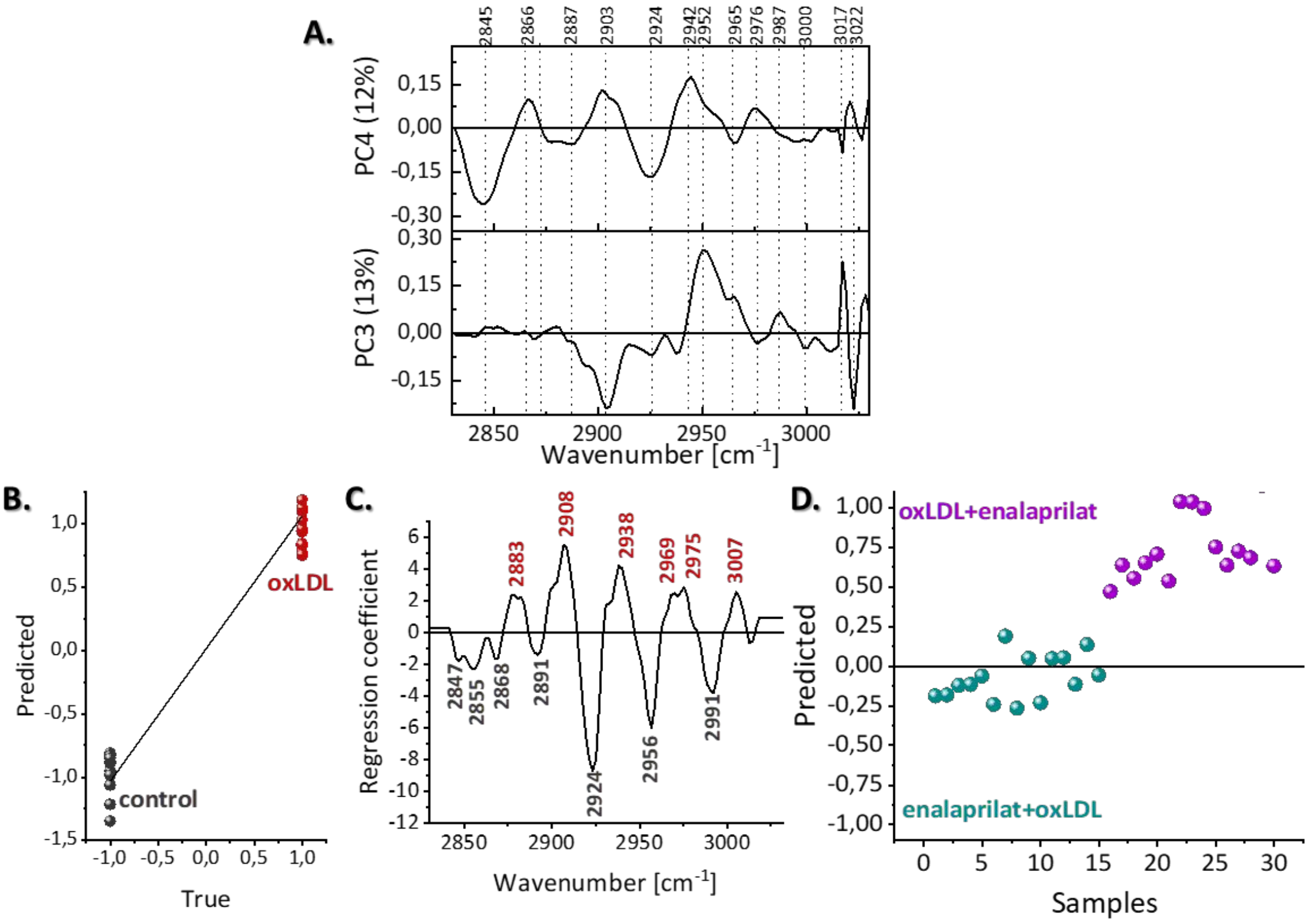

Fig. S6 Chemometric analysis of spectra from the lipid class of cells treated with enalaprilat and oxLDL. A. *PCA loading plot performed in C-H stretching region (2830 – 3030 cm^{-1}) of control (black), oxLDL (red), pretreated (enalaprilat+oxLDL, green), and post-treated cells (oxLDL+ enalaprilat, violet) for lipid class.* ***B.*** *PLS model (cytoplasm class) trained in the C-H stretching region on control and oxLDL samples.* ***C.*** *The regression coefficient plot for the class. D.* *Validation for the spectra from pretreated (enalaprilat+oxLDL) and post-treated (oxLDL+enalaprilat) cells from the cytoplasm class.*

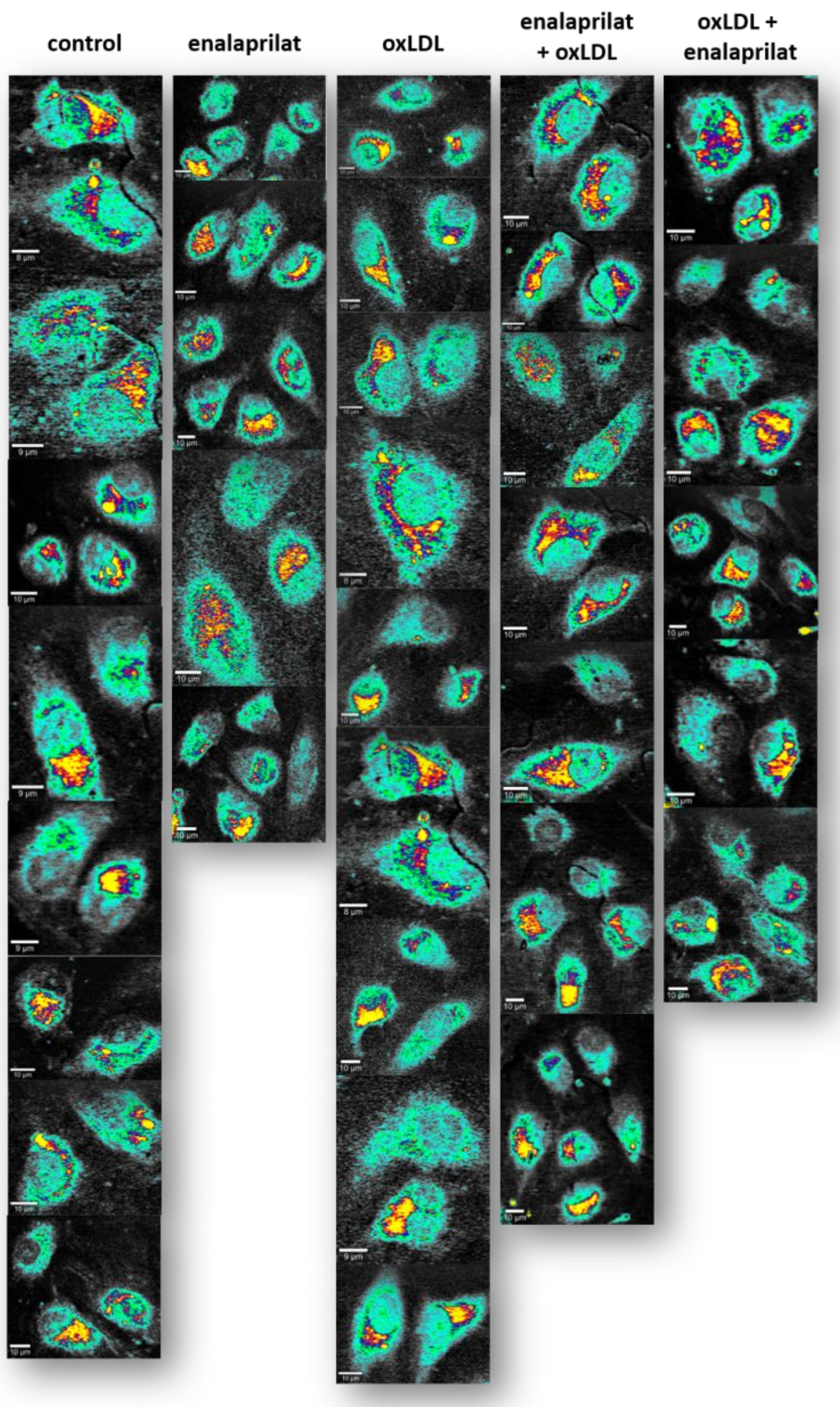

Fig. S7. Ratiometric Raman imaging of intracellular lipid distribution *(SRS intensity ratio $I_{2850\ cm^{-1}}$ / ($I_{2850\ cm^{-1}}$ + $I_{2930\ cm^{-1}}$)).*

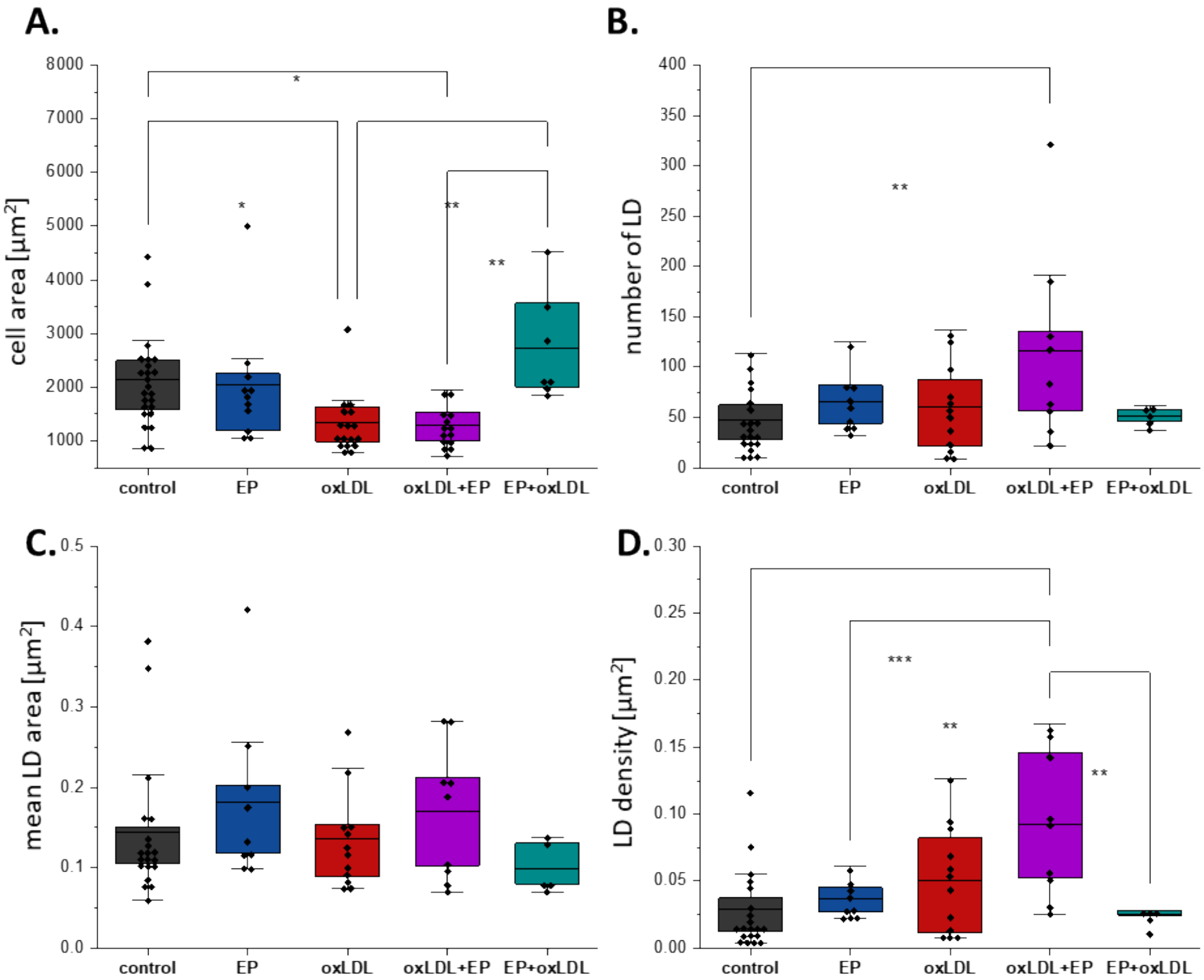

***Fig. S8 Results of LD characterization with CARS microscopy with ANOVA results. A** The cell area from manual ROI segmentation of a single cell. **B** The number of droplet-like structures within the cell area resulting from particle analysis. **C** The mean area of LDs within the cells. **D** The number of LDs is normalized to the measured cell area. Results are presented as box plots (mean ± SD, whiskers indicate minimum/maximum, line indicates the mean value), ***p≤ 0.001, **p≤ 0.01, *p≤ 0.05.*
